# High resolution biosensor to test the capping level and integrity of mRNAs

**DOI:** 10.1093/nar/gkaa955

**Published:** 2020-11-05

**Authors:** Ignacio Moya-Ramírez, Clement Bouton, Cleo Kontoravdi, Karen Polizzi

**Affiliations:** Department of Chemical Engineering, Imperial College London, London SW7 2AZ, UK; Imperial College Centre for Synthetic Biology, Imperial College London, London SW7 2AZ, UK; Department of Infectious Disease, Imperial College London, London W2 1NY, UK; Department of Chemical Engineering, Imperial College London, London SW7 2AZ, UK; Department of Chemical Engineering, Imperial College London, London SW7 2AZ, UK; Imperial College Centre for Synthetic Biology, Imperial College London, London SW7 2AZ, UK

## Abstract

5′ Cap structures are ubiquitous on eukaryotic mRNAs, essential for post-transcriptional processing, translation initiation and stability. Here we describe a biosensor designed to detect the presence of cap structures on mRNAs that is also sensitive to mRNA degradation, so uncapped or degraded mRNAs can be detected in a single step. The biosensor is based on a chimeric protein that combines the recognition and transduction roles in a single molecule. The main feature of this sensor is its simplicity, enabling semi-quantitative analyses of capping levels with minimal instrumentation. The biosensor was demonstrated to detect the capping level on several *in vitro* transcribed mRNAs. Its sensitivity and dynamic range remained constant with RNAs ranging in size from 250 nt to approximately 2700 nt and the biosensor was able to detect variations in the capping level in increments of at least 20%, with a limit of detection of 2.4 pmol. Remarkably, it also can be applied to more complex analytes, such mRNA vaccines and mRNAs transcribed *in vivo*. This biosensor is an innovative example of a technology able to detect analytically challenging structures such as mRNA caps. It could find application in a variety of scenarios, from quality analysis of mRNA-based products such as vaccines to optimization of *in vitro* capping reactions.

## INTRODUCTION

mRNAs are central molecules of life, acting as intermediates between the information stored in the DNA and the functions carried out by proteins. Despite their importance, traditionally therapeutic approaches have not considered nucleic acids as a treatment option. However, this trend has shifted during the last two decades, with the direct therapeutic use of nucleic acids, and in particular mRNAs, attracting considerable scientific and economic attention. For example, there are already several approved RNA-based drugs and many others undergoing pre-clinical and clinical trials (1,2). In parallel, research is focused on increasing the translational activity, molecule delivery into cells, and the modulation of the immune response to these drugs (3–6). Therefore, it is expected that nucleic acid-based drugs will gain increased application as highly targeted therapeutic treatments applied to, e.g. vaccination or cancer therapy (7,8).

This new group of drugs will need to be accompanied by adequate quality control tools, from both a large-scale production and point-of-care perspective (9). A key factor for quality control is the integrity of the nucleotide chain, which is of particular importance for RNAs since they are sensitive to degradation. There are a number of well-known options for structural and integrity analyses of RNAs. These are based, for example, on absorbance assays, molecular mobility, template amplification, complementary oligonucleotide hybridization, aptamer probes, or protein-RNA interactions. Signal outputs of these assays include fluorescence, light refraction/transmission, and current production, among others (10,11). In this regard, biosensors (sensing devices that incorporate a biological recognition element) are ideal for quality control of molecules such as mRNAs, since they offer high specificity and large dynamic ranges, enabling detection limits down to single molecules (12). Techniques such as fluorescence *in situ* hybridization (FISH), aptamers or molecular beacons are commonly used for the *in vivo* visualization of mRNAs (13–15). However, there are some challenges remaining in the detection of mRNA such as secondary structures or low mobility of long chains (11), which can be overcome using biosensors. For example, Li *et al.* (16) developed a biosensor based on quantum dots and the action of a duplex-specific nuclease, and Huertas *et al.* (17) proposed a detection system based on surface plasmon resonance for the analysis of mRNA fragments. Moreover, Kindt *et al.* (18) proposed a biosensor able to analyse complete mRNAs, based on a bead-based silicon photonic resonator in conjunction with chaperone probes to reduce the mRNA secondary structure and enhance the access to the target. There are also biosensors focusing on the detection of specific miRNAs and mRNAs or even multiplexed detection of nucleic acids that have a great interest for diagnosis (19–21).

In the particular case of mRNAs transcribed *in vitro*, quality control must be able to assess several mRNA features essential for functionality *in vivo*, such as 5′ end capping, polyadenylation and integrity. mRNAs transcribed by eukaryotic RNA polymerase II undergo a co-transcriptional modification on their 5′-end known as capping (22,23). The most common 5′ end cap structure, known as *cap 0*, involves the addition of a 7-methyl guanosine group to the first nucleoside of the chain through a 5′-5′ triphosphate bridge (m^7^GpppN). This cap structure is relevant for nuclear export, mRNA processing and translation, and also helps to prevent 5′ exonucleolytic degradation (24–27). Polyadenylation of the 3′ end of the transcript is the second major mRNA modification. It is linked with the cap structure in some functions such as translation initiation, and is also relevant for RNA splicing and half-life (26). Therefore, the presence of these modifications on mRNA drugs are necessary to bypass the cellular quality control mechanisms, prevent premature mRNA degradation, increase translational yield and/or avoid immunogenicity, factors that can severely affect the efficacy of the treatment (2,28,29).

Despite the importance of the 5′ cap structures for mRNA performance, to date, no biosensors have been designed to detect the 5′ cap. There are, however, some well-established analytical techniques that can be used for analysis. On one hand, there are simple methods that would be accessible for any standard laboratory such as enzymatic digestion-based approaches that make use of the combined action of an RNA 5′ polyphosphatase and a 5′ to 3′ ribonuclease to remove uncapped RNA and estimate the percentage capping of a sample. However, the accuracy of digestion-based methods is limited. In addition, they can present problems such as enzyme stalling on secondary structures (30,31) or non-5′-monophosphate-specific activity of the ribonuclease. Such problems can lead to over- or underestimates of the capped fraction of the mRNA sample.

Another example is gel electrophoresis, although this can only be used with short RNAs unless it is combined with a cap-affinity purification step (27,32,33) or additional enzymatic treatments (34). On the other hand, more sophisticated methods can be used at the expense of being more labour and equipment intensive and therefore less accessible. Surface plasmon resonance, fluorescence quenching or NMR have also been used (27,35,36), but most of these techniques lack the level of detail required for an accurate estimation of the capping status of an mRNA. More modern techniques such as RNAseq are gaining importance, and variations of this, such as CAGE (Cap Analysis Gene Expression), rely on the presence of the cap to obtain full length cDNAs, so they can be used to study the capping status of mRNA pools (37,38). Similarly, Blewett *et al.* proposed another alternative based on a splinted ligation and quantitative reverse transcription PCR assay able detect uncapped RNAs, which is to date, the only available procedure to accurately quantify the capping level of mRNAs (39). Finally, LC–MS has also been used for the detection of cap structures (9,40). However, all these approaches have the common characteristic of requiring sophisticated equipment and chemical modification or extensive treatment of the mRNAs, limiting their applicability for quality control. Most of these techniques are applied to transcriptomic studies where intricate protocols or access to high-tech equipment is not a bottleneck. Conversely, in this work we aim to bridge the existing gap between these highly sophisticated methodologies and those available for non-specialised end-users. We focused on the particular case of mRNAs synthesized *in vitro* for medical applications, where both the integrity and capping level must satisfy a quality threshold prior to their administration. Here we present a biosensor designed to test these two parameters in a single step. The design followed the principles of flexibility, ease of application and low technological requirements, making it suitable for both field applications and in industry.

**Figure 1.**
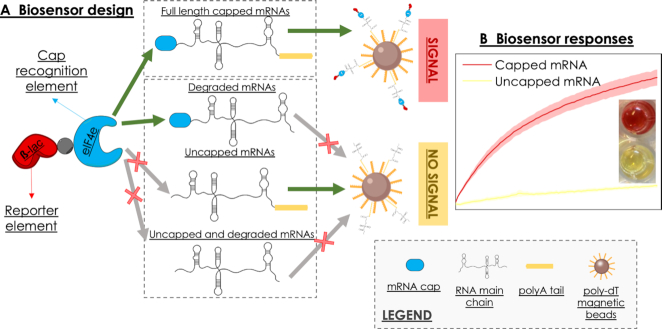
(**A**) Elements of the B4E biosensor. The B4E chimeric protein is composed of a cap recognition element (murine eIF4E) and a reporter element (β-lactamase). Capped and non-degraded mRNAs (top) will bind to the pdT_25_ functionalised magnetic beads by annealing via the polyA tail. At the same time, the B4E peptide will recognise and bind the cap on the 5′ end of the mRNA molecule. Uncapped or degraded mRNAs (bottom) will not bind to the beads and/or B4E, therefore, at least one of the elements of the sensor will be missing in the colorimetric assay. (**B**) Response of the biosensor, where a change in colour is detected due to the hydrolysis of nitrocefin by the action of the β-lactamase only when capped and full-length mRNA has been loaded.

The biosensor uses an engineered chimeric protein (B4E, a fusion of the murine eIF4E protein and ß-lactamase) in conjunction with poly-deoxythymidine oligonucleotide-functionalised beads to simultaneously probe for both polyadenylation and 5′ capping. In the first step, the mRNA to be analysed anneals to the beads via the polyA tail. Subsequently, the murine eIF4E domain in the B4E protein binds to those mRNAs with a m^7^G cap structure, and finally the presence of mRNA-bound B4E protein is colorimetrically tested via the β-lactamase domain. If the RNA is degraded, either the cap structure or the polyA tail (or both) will be missing, blocking the signal of the biosensor (Figure 1). The proposed system constitutes a low-tech approach to detect the integrity and capping status of mRNAs in a single step, bypassing the need for labelling, amplification or physicochemical modification of the nucleotide chain. Moreover, the main feature of our biosensor, and what differentiates it from existing approaches, is the combined detection of both ends of the RNA chain. Combined with the specific recognition of the cap structure, our method allows the simultaneous validation of the capping status and integrity. The flexibility and ease of standardization of this approach makes it applicable to a broad scope of uses, from in-depth transcription studies to quality control application at industrial and end-user levels.

## MATERIALS AND METHODS

### Chemicals

Common use chemicals such as LB media and salts, β-d-1-thiogalactopyranoside (IPTG), dithiothreitol (DTT), bovine serum albumin (BSA), phenylmethylsulfonyl fluoride (PMSF), wheat germ tRNA and 2-mercaptoethanol (2-ME) were analytical grade and purchased from Sigma Aldrich (UK). Tween-20 was supplied by Fisher Scientific (UK). Nitrocefin was purchased from Thermo Scientific (UK). Oligonucleotides where synthetized by Invitrogen (UK). Restriction enzymes were obtained from New England Biolabs (NEB, UK).

### Plasmid construction

The B4E fusion protein was synthetized by GeneArt (Thermo Scientific) and consists of the *amp^R^* gene (encoding the enzyme β-lactamase without the native signal sequence) fused to residues 28–217 of the murine eIF4E incorporating the K119A mutation, which is known to increase the cap binding affinity of the protein (32,41,42). The two domains of the fusion protein were connected by a Gly-Ala_x10_ linker and a His_6_ tag was also included on the N-terminus. The sequence was codon-optimized for expression in *E. coli* (see supplementary information for the DNA sequence and Supplementary Table S2 for oligonucleotide sequences used in cloning).

A set of DNA templates for *in vitro* transcription of mRNAs for the biosensor tests were constructed based on the plasmid pRSET-A (Thermo), which contains a T7 promoter and transcription terminator. A 50 nt synthetic polyA tag was introduced into a pET28a+ plasmid between the *Not*I and *Xho*I restriction sites to generate pET28a+-polyA and this was moved to the pRSET-A plasmid using the *Xba*I and *Not*I restriction sites, creating pRSET-T1. A similar procedure was used to prepare DNA templates 2 and 3 to create the plasmids pRSET-T2 and pRSET-T3, that incorporate extra nucleotides upstream the polyA tail by the integration of the *lac*I gene and a *NtGnT*I genes into the pRSET-polyA plasmid, respectively.

*Escherichia coli* DH5α (NEB) was used for the cloning work. All the constructs were sequence-verified by Eurofins Genomics (Ebersberg, Germany). Qiagen miniprep kit (UK) was used for all plasmid purifications. The DNA Clean & Concentrator kit (Zymo Research, UK) was used for purification of linear DNA fragments.

### mRNA sample preparation

#### *In vitro* mRNA synthesis

Capped and uncapped mRNA molecules were prepared by *in vitro* transcription. DNA plasmid templates were linearized by restriction digestion with *Xho*I, which cuts directly downstream of the polyA sequence. The linearized plasmids pRSET-T1, pRSET-T2 and pRSET-T3 encoded for RNA_1_, RNA_2_ and RNA_3_ with sizes of 254 nt, 1051 nt and 2699 nt, respectively (their sequences are provided in supplementary material). For the uncapped versions of the mRNAs, the *in vitro* transcription reactions were carried out with 1 μg of DNA template, 3 μl of each NTP at 100 mM, 400 units (2 ul) of T7 polymerase (Thermo, UK), 80 U of RNase inhibitor (NEB) and nuclease free water to final volume of 200 μl. The reaction was incubated at 37°C overnight. The DNA template was removed by digestion with of 5 μl of DNAseI (NEB) at 37°C for 1 h. For the synthesis of capped RNA, an anti-reverse cap analogue (ARCA) was co-transcriptionally incorporated using the HiScribe™ T7 ARCA kit (NEB) and the same templates, giving rise to cRNA_1_, cRNA_2_ and cRNA_3_, respectively. For both syntheses, RNA Clean & Concentrator kit (Zymo Research) was used for purification of the transcribed RNAs. In parallel, a mRNA-based vaccine under development (mRNAv, 6507 nt) was tested. It was also transcribed with a T7 RNA polymerase from a linear DNA template using the MEGAscript^®^ system (Invitrogen) at 37°C for 2 h. mRNAv was post-transcriptionally capped (with the ScriptCap cap 1 capping system (CellScript™, USA) where the ScriptCap 2'-*O*-methyltransferase was omitted, and polyadenylated with an *E. coli* Poly(A) Polymerase (NEB). In between each step the RNA was purified by LiCl precipitation. The yield and purity of all *in vitro* transcribed RNAs is shown in Supplementary Table S1 and Supplementary Figure S2. Since mRNAv is under development, only the first 200 nt of the 5′-UTR have been provided in this work (see supplementary material). The capping efficiency for all these transcripts was estimated by a sequential digestion with RNA 5′ polyphospatase and a 5′ to 3′ ribonuclease (supplementary information, section 1.5). The capping efficiency was between 80 and 90% for all samples (see Supplementary Figure S3 for details).

#### mRNA extraction from mammalian cells

mRNA from Chinese hamster ovary (CHO) cells was extracted from a CHO-S cell line (Thermo) at days 2, 4 and 8 of batch culture. Cells were grown in 50 ml of CD-CHO culture media (Gibco, Thermo) supplemented with 8 mM L-Glutamine and 1x hypoxanthine/thymidine supplement (Gibco, Thermo), in 250 ml flasks at 37°C with 5% CO_2_ and shaking at 120 rpm. After revival, cells were subcultured twice every 72 h before starting the batch culture with a seeding density of 3·10^5^ cells/ml. Cells were sampled from the culture at the specified times, pelleted by centrifugation at 400 g and 4°C for 5 min and washed once with ice-cold PBS. Cell pellets were preserved in RNAlater (Thermo) at −20°C until mRNA extraction using the mRNA direct extraction kit (NEB). The procedure indicated by the manufacturer was followed, with some modifications. The cell number was increased to 10^6^ or 2.33 × 10^6^ cells per 100 μl of magnetic beads. After cell lysis, a protease digestion step was included to reduce protein carryover during the purification, using 2.5 μl of enzyme (proteinase K > 500 u/ml, Thermo) per ml of lysate and incubating for 15 min at 55°C. Finally, RNase-free water was used instead of elution buffer. Extracted samples were further purified and concentrated with RNA Clean & Concentrator kit (Zymo Research). A modified method was used to extract the total RNA from cells to be used for biosensor control experiments (supplementary methods).

#### RNA refolding

Before being tested, mRNAs were refolded to recover their tertiary structure, adapting the protocol suggested by Cantara *et al.* (43). The mRNA samples were defrosted on ice, and diluted in Buffer A (50 mM HEPES, 100 mM KCl, pH 7.4). The mixture was incubated at 80°C for 2 min followed by 60°C for 2 min. MgCl_2_ was added to a final concentration of 1 mM followed by a final incubation at 37°C for 30 min.

### Protein production, purification and validation

The plasmids pET28a-B4E-v.i, pET28a-B4E-v.ii or pET28a-βLac, used to express B4E-vI, B4E-vII and the β-lactamase control proteins respectively, were transformed into chemically competent *E. coli* BL21 (DE3) cells. An inoculum culture was grown overnight at 30°C and 250 rpm in lysogeny broth (LB) medium containing 50 μg/ml of kanamycin. Subsequently, 200 ml of medium in a 2 l flask were inoculated with 1.25% (v/v) of the initial culture. Cells were grown at 25°C and 250 rpm to OD600 of 0.5. Protein expression was induced by adding IPTG to a final concentration of 0.5 mM. After 16 h of growth at 25°C, cells were pelleted by centrifugation at 4°C for 20 min at 4000 g and resuspended in 20 ml of lysis buffer (50 mM NaH_2_PO_4_, 1 M NaCl, 20 mM imidazole, 0.1% Triton-100X and 10 mM 2-ME and 5% glycerol, pH 8). The mix was supplemented with 1 mM PMSF and 1 mg/ml of lysozyme (final concentrations) and incubated for 30 min at 4°C. The mix was divided into two 15 ml Falcon tubes where cells were disrupted by sonication for 140 s (10 s ON/ 15 s OFF cycles, 65% amplitude) at 4°C with a sonicator equipped with a ¼’ probe (FB 120, Fisher Scientific). The insoluble fraction of the lysed cells was pelleted by centrifugation at 16,000xg for 30 min at 4°C. The soluble fraction was incubated with 1 ml of 50% Ni-NTA agarose resin (Qiagen) for 1 h at 4°C with gentle agitation. After that, the sample was divided into two aliquots and packed into two disposable polypropylene columns. Each was washed twice with 4 ml of wash buffer (50 mM NaH_2_PO_4_, 1 mM NaCl, 50 mM imidazole, 5% glycerol, pH 8). Finally, the protein was recovered in 2 elution fractions of 0.3 ml each with elution buffer (50 mM NaH_2_PO_4_, 1 mM NaCl, 5% glycerol, pH 8) at 75 mM imidazole, followed by three additional elution fractions of 0.3 ml with elution buffer containing 250 mM imidazole. Supplementary Figure S1 shows the SDS-PAGE analysis of the various fractions during purification. Elution fractions 2, 3 and 4 were combined and buffered-exchanged using a centrifugal column concentrator with a 30 kDa MWCO for B4E or a 10 kDa MWCO for β-lactamase (Vivaspin 20, GE Healthcare, UK). Here, 1 ml of protein buffer (HEPES 50 mM, KCl 100 mM, 10% v/v glycerol and pH 7.2) was run through the column three times, and the final volume reduced to 200 μl.

#### m^7^GTP-functionalised agarose test

The ability of B4E fusion protein to bind cap structures was assayed using m^7^GTP-functionalished agarose resin. 50 μl of γ-aminohexyl-m^7^GTP-agarose resin (Jena Bioscience, Germany) was equilibrated with three washes of 100 μl binding buffer (HEPES 50 mM, KCl 100 mM, glycerol 10%, pH 7.4) and resuspended in 60 μl of binding buffer. 40 μl of protein at a concentration of 14.5 μM was added, followed by incubation on ice for 1 h. The resin was washed with 100 μl of binding buffer 4 times. Finally, the bound protein was eluted using protein buffer supplemented with 500 μM of m^7^GTP in three fractions of 100 μl. For each elution, the resin was incubated for 5 min prior to centrifugation for 2 min at 600 g in a bench centrifuge (Eppendorf Minispin, UK) and removal of the supernatant. βLac was used as a negative control. The presence of either B4E or βLac was tested in the three elution fractions, and in the resin before and after the elution steps with a nitrocefin assay using 5 μl of each fraction (see below).

#### Biolayer interferometry

Biolayer interferometry (BLI) was used to study the interaction between RNAs and B4E. An interferometer (BLItz^®^, ForteBio, USA) equipped with high precision streptavidin sensors (SAX, ForteBio) was used. The sensors were hydrated with buffer A (HEPES 50 mM, KCl 100 mM, pH 7.4) for at least 10 min and no more than 1 hour before being used and kept submerged until the beginning of the measurement. The BLI assay was composed by the following steps: (i) sensor equilibration for 30 s with 250 μl of buffer A; (ii) binding of biotinylated pdT_25_ oligonucleotide for 1 min using 250 μl of a 0.1 μM solution in buffer A; (iii) wash with 250 μl of buffer A for 30 s; (iv) binding of the refolded RNA using 4 μl of 0.5 μM RNA for 2 min; (v) wash with 250 μl of buffer B (HEPES 33 mM, KCl 66 mM, 0.1% BSA, 0.1% Tween20, 6 mM DTT, pH 7.4) for 30 s; (vi) protein binding with 250 μl of 0.45 μM B4E in buffer B for 4 min; (vii) desorption with 250 μl of buffer B for 2 min.

### Biosensor assay

Biosensor assays are composed of the following steps: (i) 10 μl of streptavidin-coated magnetic beads (Dynabeads T1, Thermo) were washed three times with 20 μl of buffer A (HEPES 50 mM, KCl 100 mM, pH 7.4) and resuspended in 20 μl of 2× buffer; (ii) 20 μl of biotinylated poly-deoxythymidine oligonucleotide (pdT_25_) at 3.75 μM in RNase free water were added to the beads and incubated for 10 min; (iii) the beads were washed with 20 μl of buffer A; (iv) 20 μl of 0.6 μM refolded mRNA were added and incubated for 20 min; (v) beads were washed with 100 μl of buffer B (HEPES 33 mM, KCl 66 mM, 0.1% BSA, 0.1% Tween20, 6 mM DTT, pH 7.4) for 5 min; (vi) 50 μl of 0.45 μM B4E in buffer B were added and incubated for 1 h; (vii) unbound protein was removed with three washes with 200 μl of buffer B; (viii) beads were resuspended in 200 μl of buffer A, and 6 μl (equivalent to 3 μg of beads) were used for the nitrocefin assay. All the required separation steps were performed in a magnetic rack and the incubations at room temperature on a rotatory platform at 60 rpm. For the longest mRNA transcripts (cRNA_3_, mRNA_v_ and mRNA_CHO_), conditions were adjusted in order to maintain the dynamic range of the biosensor and are indicated in each case in the results and discussion section below.

#### Nitrocefin colorimetric assay

The presence of B4E in several samples such as magnetic beads, m^7^GTP resin or elution fractions was tested using a nitrocefin hydrolysis assay (Supplementary Figure S4). The tests were performed in a 96-well plate. Nitrocefin solution (0.1 M nitrocefin, 50 mM phosphate buffer, pH 7) was added to the sample to a final volume of 200 μl. The spectroscopic response of the biosensor over time was followed by absorbance measurements at 492 nm with a CLARIOstar plate reader (BMG Labtech, UK), measuring every minute for 90 min. Reads at 1200 s of reaction were used to calculate the transfer functions for cRNA_1_, cRNA_2_ and cRNA_3_. All experiments were performed in triplicate.

## RESULTS AND DISCUSSION

### Validation of the recombinant protein B4E

The proposed biosensor is based on a fusion protein, B4E, that combines the abilities to recognise and bind to the m^7^G structures on mRNAs with colorimetric reporter properties. To construct B4E, the enzyme β-lactamase without the signal sequence was fused to residues 28–217 of the murine eIF4E initiation factor (44). eIF4E is the first element of the 4E initiation complex to bind to mRNAs (45,46), and tagged versions of the protein have also been used for purification of capped RNA (27). Prior to its use, the cap-recognition and reporting properties of the chimeric protein B4E were tested.

#### Activity of the eIF4E region of the recombinant protein

A m^7^GTP-functionalised agarose resin was used to validate the functionality of the cap binding region of B4E. The presence of B4E in either the resin or elution fractions was tested using a nitrocefin colorimetric assay. As shown in Supplementary Figure S5a, B4E binds to the m^7^GTP-functionalised resin, as suggested by the fast hydrolysis of the nitrocefin upon its incubation with the resin. Most of the protein was eluted from the resin after three washes with m^7^GTP (Supplementary Figure S5a and b). In contrast, a control experiment with β-lactamase showed no detectable amount of protein bound to the beads or in the fractions eluted with m^7^GTP (Supplementary Figure S5c and d). These results indicate that the resin-protein interaction is based on recognition of the m^7^GTP group by B4E and confirm that the cap binding and ß-lactamase regions remain functional in the fusion protein. Various optimisation steps were undertaken to assess the effects of pH, buffer composition, etc on biosensor function (Supplementary Figures S6–S11).

#### Biolayer interferometry (BLI)

**Figure 2.**
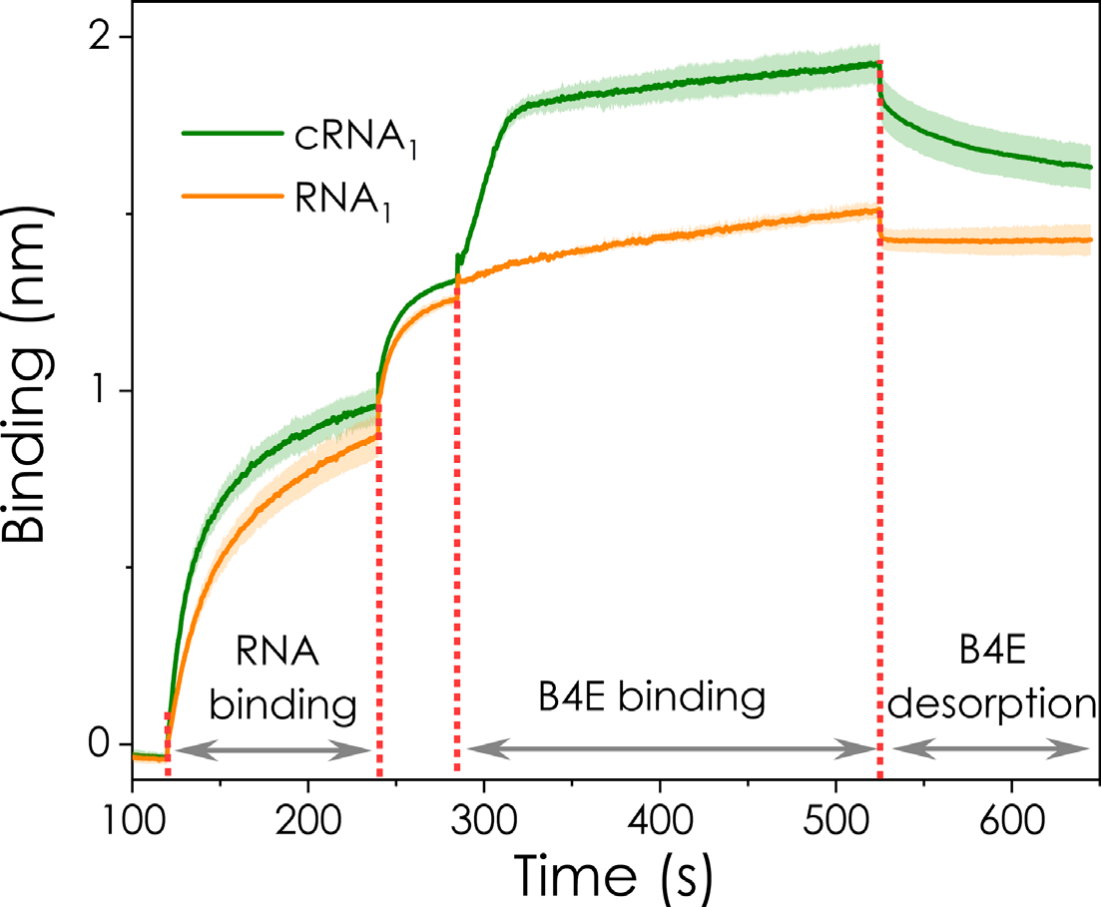
Bio layer interferometry (BLI) tests. Capped (cRNA_1_) and uncapped (RNA_1_) versions of RNA_1_ were assayed. Three different molecular interaction stages are depicted for both: (i) RNA loading and binding to the pdT_25_ oligo-functionalised sensor, (ii) B4E protein loading and (iii) desorption of B4E in buffer B. Both RNAs clearly bind to the pdT_25_-functionalised sensor. However, mRNA-B4E binding is only detected for the capped version of the mRNA (cRNA_1_), which is also reflected in the differences on the desorption steps for both mRNAs. Averages and standard deviation of three experimental replicates are shown. For clarity purposes, the signals from the loading of the pdT_25_ oligo on to the interferometer sensor have been omitted and only the results from the RNA binding step onwards are shown. The intermediate increase in the signal between steps (i) and (ii) corresponds to an equilibration step with buffer B.

Biolayer interferometry experiments provided a more detailed insight into the interaction of B4E with mRNA and its cap structure via direct detection of the molecular interactions. The streptavidin sensor was initially functionalised with pdT_25_ oligonucleotides and then loaded with either capped or uncapped mRNAs. As shown in Figure 2, during the first binding step both mRNAs bound similarly to the sensor, showing that their polyA tails are equally accessible. B4E was then loaded in a second step. In contrast to the mRNA binding, the signal clearly differed, showing a fast association of protein and mRNA only if capped mRNA (cRNA_1_) is previously loaded on the sensor. The initial association was followed by a slower and constant increase in the signal, which is also observed for uncapped mRNA (RNA_1_), likely due to a weaker non-specific binding that increases over time along the association step. During the final desorption step with buffer B, the signals also differ for capped and uncapped RNA. While for RNA_1_ only a rapid decrease takes place, the desorption curve for cRNA_1_ shows the same rapid initial decay followed by a slower decrease in the signal. Similarly, this can be attributed to an initial desorption of the weak non-specifically interacting protein, followed by the slower desorption process of the cap bound B4E. Analogous patterns have been reported in surface plasmon resonance experiments with eIF4E (47). Hence the BLI results show that the ability of the murine eIF4E initiation factor to recognise and bind mRNA cap 0 structures has been preserved in the fusion protein.

### Cap biosensor studies

#### Biosensor use for qualitative studies of the mRNA integrity

Once the functionality of B4E as the combined sensing and reporting element of the biosensor was confirmed, we tested the ability to qualitatively and quantitatively analyse the integrity and capping levels of mRNAs. According to the biosensor design, the mRNAs must fulfil both quality criteria of having a 5′ cap and polyA tail, and thus be fully intact, in order to trigger the response of the biosensor (Figure 1). If mRNAs are uncapped or undergo cleavage or degradation, one of these features will be missing, leading to no response.

**Figure 3.**
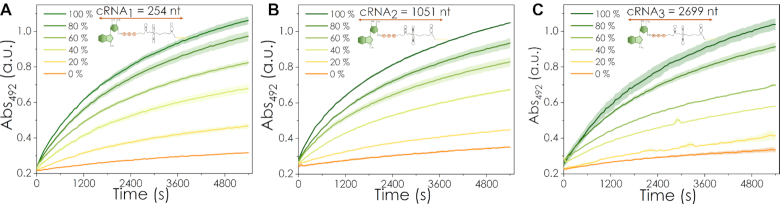
Biosensor responses at different percentages of capped mRNAs. Spectroscopic responses of the biosensor over time when loaded with different percentages of capped mRNAs, while maintaining the total concentration of mRNA constant. Figures A, B and C correspond to mRNAs of different lengths: 254 nt (cRNA1), 1051 nt (cRNA2) and 2699 nt (cRNA3), respectively. For cRNA1 and cRNA2 the standard procedure described on the methods section was followed. For cRNA3 the working concentration used was 0.3 μM, and 4 μg of beads were used for the assay. Averages and standard deviations of three experimental replicates are shown.

The biosensor was initially tested with three mRNAs of different lengths (cRNA_1_: 254 nt, cRNA_2_: 1051 nt and cRNA_3_: 2699 nt) that were co-transcriptionally capped with an ARCA oligonucleotide. Each was mixed in different proportions with their respective uncapped versions (RNA_1_, RNA_2_ and RNA_3_), while maintaining the total RNA concentration constant. For all three mRNAs, the response of the biosensor correlated with the percentage of capped RNA as shown in Figure 3. Significantly, the proposed system is not only suitable for a yes/no analysis of whether a mRNA is capped and intact but is also sufficiently sensitive to distinguish between different capping/degradation ratios. This makes it possible to build a transfer function correlating the percentage of capped RNA and the signal, enabling the relative quantification of full-length and capped mRNA in a sample. To create the transfer function, the initial (linear) slope of the enzymatic hydrolysis of nitrocefin was used, and was taken as the time frame where linear regression of the absorbance has an *R*^2^ > 0.98. In this regime, the amount of capped mRNA can be directly related to the amount of hydrolysed nitrocefin, i.e. the change in colour/absorbance. Interestingly, the signal-to-noise ratio was almost unaltered in the presence of NTPs and the DNA template used for the transcription reaction (Supplementary Figure S6), which could be of special interest for monitoring mRNA quality and purity in the development of large-scale synthesis and downstream processing routes for mRNA manufacture.

**Figure 4.**
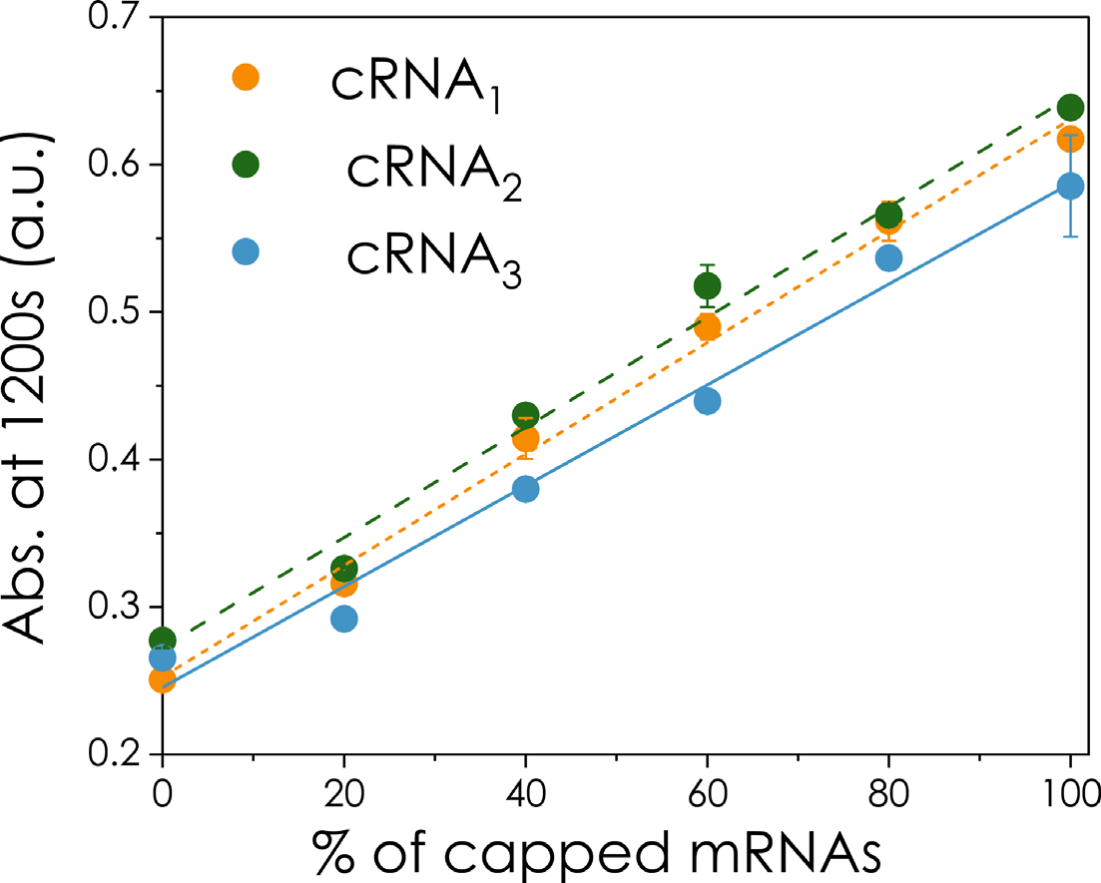
Transfer function of the response of the sensor against different percentages of cRNA_1_, cRNA_2_ and cRNA_3_ at 1200 s, where the nitrocefin hydrolysis was still within the first-order (linear) reaction phase. The graph shows that the response of the sensor is highly linear across the whole range of capping percentage and the dynamic range remains almost unaltered for a 10-fold increase in the size of the analysed mRNA (cRNA_1_ versus cRNA_3_). The parameters of the transfer functions are indicated in Supplementary Table S3, the *R*^2^ values are above 0.984 in all the cases, denoting the high reproducibility of the assay and the linearity of the biosensor response during this time range. Error bars represent the standard deviation of three replicates.

Figure 4 shows that the transfer function after 20 min of reaction has a linear response for the whole range of capped RNAs, a feature of special relevance for low-tech applications where visual inspection might be used as a readout (see *Visual assessment of the integrity and capping levels* *of mRNA* below). Notably, the transfer functions also show that the biosensor retains its sensitivity over a wide range of mRNA sizes, with more than a 10-fold increase in the chain length from cRNA_1_ (254 nt) to cRNA_3_ (2699 nt). Also, inserting an aptamer that produces strong secondary structure into the 5′ region of cRNA_1_ did not affect the biosensor response (Supplementary Figure S7). Therefore, within the range tested, the biosensor is unaffected by one of the main challenges related to the detection of large and/or highly structured mRNA molecules, i.e. the orientation or steric hindrance of the target nucleotide/structure (11). The high signal-to-noise ratio (SNR) can be attributed to the fact that the proposed system relies on the 5′ cap and polyA tail structures on mRNAs, which are at the termini, and therefore more accessible. In contrast, techniques involving a probe or protein recognition of inner nucleotide structures such aptamers can be more dependent on the local folding of the target mRNA in that region and its accessibility, as was the case in our previous study using an RNA aptamer (48). Targeting the termini of the mRNA can also explain why the sensitivity is preserved across a wide range of mRNA lengths, suggesting that the B4E biosensor could be applicable for a broad variety of mRNA transcripts, with only minor adaptation required for each particular case.

One of the main advantages of the B4E biosensor is that it does not require the alteration of the mRNA sequence (e.g. to add recognition sequences) or further amplification reactions, in contrast to other cap detection systems (39,40). The affinity and specificity of B4E towards the m^7^G structure is a key factor as eIF4E shows binding affinities orders of magnitude higher for cap-like than for non-cap structures (24,49,50). The results in Figures 3 and 4 suggest that this specificity has been maintained in the recombinant protein B4E, as shown by the low background signal with uncapped mRNA. In addition, the high turnover rate of the β-lactamase as the reporter module and the low non-specific binding also play an important role in decreasing the limit of detection (LOD) and increasing the SNR, which means minimal amounts of mRNAs and protein are required for the assay. In total, 12 pmol of mRNA (in mass 0.89 and 3.94 μg for cRNA_1_ and cRNA_2_ respectively); 6 pmol or 5.03 μg for cRNA3, and 10 pmol of B4E and 100 μg of magnetic beads were used per assay. The B4E biosensor was able to detect variations in the capping level in increments of at least 20%. The LOD was ∼20% capped RNA, i.e. 2.4 pmol in a 12 pmol sample. This value is in line with that reported by Blewet *et al.* for their splinted ligation RT-PCR assay (LOD of 1.5 μg); while the LC-MS method proposed by Bervely *et al.* needs 100 pmol of RNA sample (9,39). It is also important to consider that 4% or less of the prepared magnetic beads are required for the colorimetric assay step, allowing for technical replicates to be completed from the same sample. In addition, since the biosensor assay is suitable for automation, the required amount of sample and protein might potentially be reduced in the future. Additional experiments on the specificity of B4E-mRNA interactions and the optimization of the experimental conditions for the biosensor are included in the supplementary material (Supplementary Figures S8 to S11).

#### Visual assessment of the integrity and capping levels of mRNA

Another key feature of the presented biosensor is its ease of application, which allows a simplification of the method to a visual inspection test if required. For example, Supplementary Figure S13 shows how the quantitative results from Figure 2A look when analysed visually. There are clear differences in appearance between samples with different percentages of capped mRNA, suggesting that the on-field testing by visual inspection for a rapid detection of a meaningful decrease in the quality of the mRNA would be possible ahead applications such as vaccination campaigns. The equipment required for the biosensor assay is minimal both for visual inspection, but also for absorbance measurements, since low-cost portable spectrophotometers are readily available. The assay is also compatible with miniaturisation or automation, where the use of magnetic beads as a platform for the assay leads to ease of handling.

**Figure 5.**
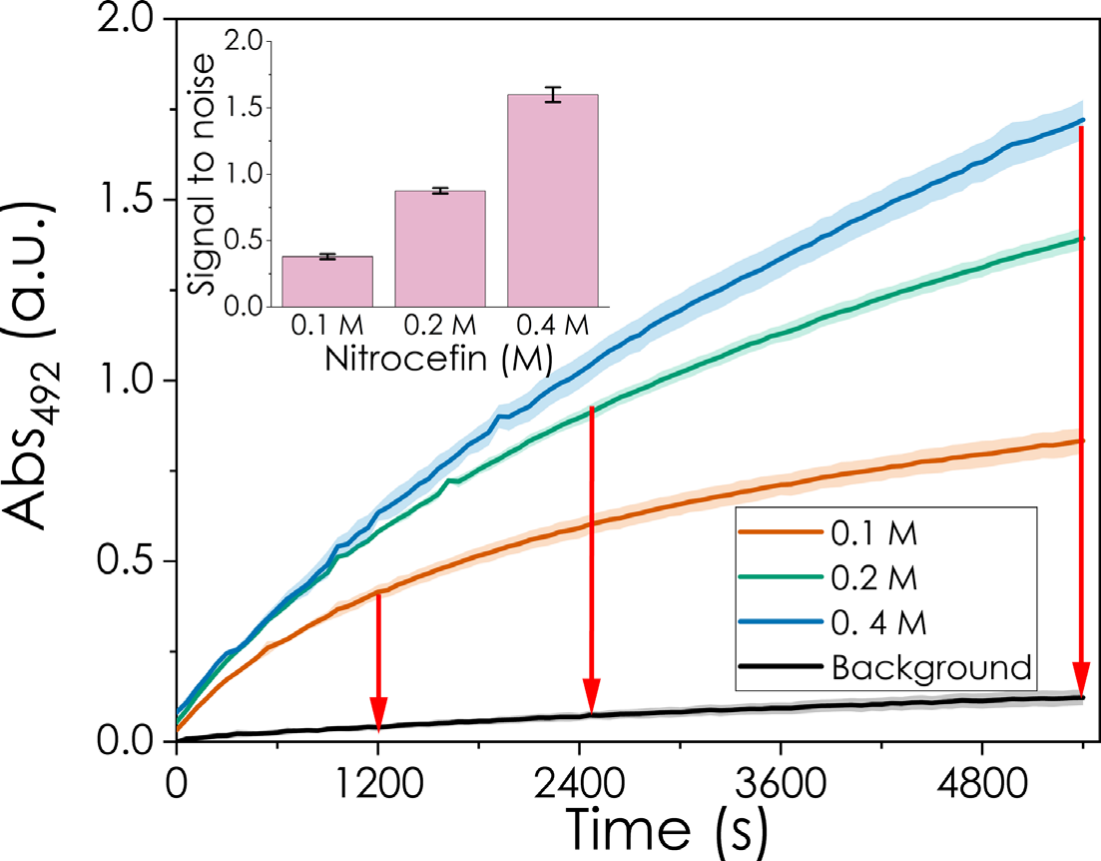
Effect of the nitrocefin concentration on the linear reaction period for biosensor assays with 100% capped cRNA_1_. Arrows indicate timepoints when the reaction is not considered linear anymore (*R*^2^ < 0.98). The inset plot corresponds to the signal-to-noise difference at the timepoints indicated by the red arrows for each nitrocefin concentration (1200, 2640 and 5400 s, respectively). Error bars represent the standard deviation of three replicates.

To further increase the applicability of the biosensor to low-tech environments, the effect of the concentration of nitrocefin was analysed. As shown in Figure 5, higher nitrocefin concentrations extended the linear range of the hydrolysis reaction, and, as a consequence, increased the dynamic range of the biosensor. The increase in dynamic range is also useful for on-field applications of the biosensor, given the lower sensitivity for discriminating between different red and yellow tones in the visual tests compared to spectrometry measurements.

#### Biosensor tests on functional mRNAs

##### mRNA-based vaccines

After demonstrating the satisfactory performance of the B4E biosensor, we aimed to explore its applicability with functional and more structurally complex mRNAs. The first test case was an mRNA vaccine currently under investigation (mRNAv). mRNAv is significantly longer compared to the previously tested transcripts (6507 nt versus the 2699 nt for cRNA_3_). Additionally, mRNAv was capped using a vaccinia capping system and thus caries a natural cap 0 structure, while the previous cRNAs used the ARCA cap analogue. Thus, this test also served to confirm the activity of B4E towards the natural cap 0 structure.

**Figure 6.**
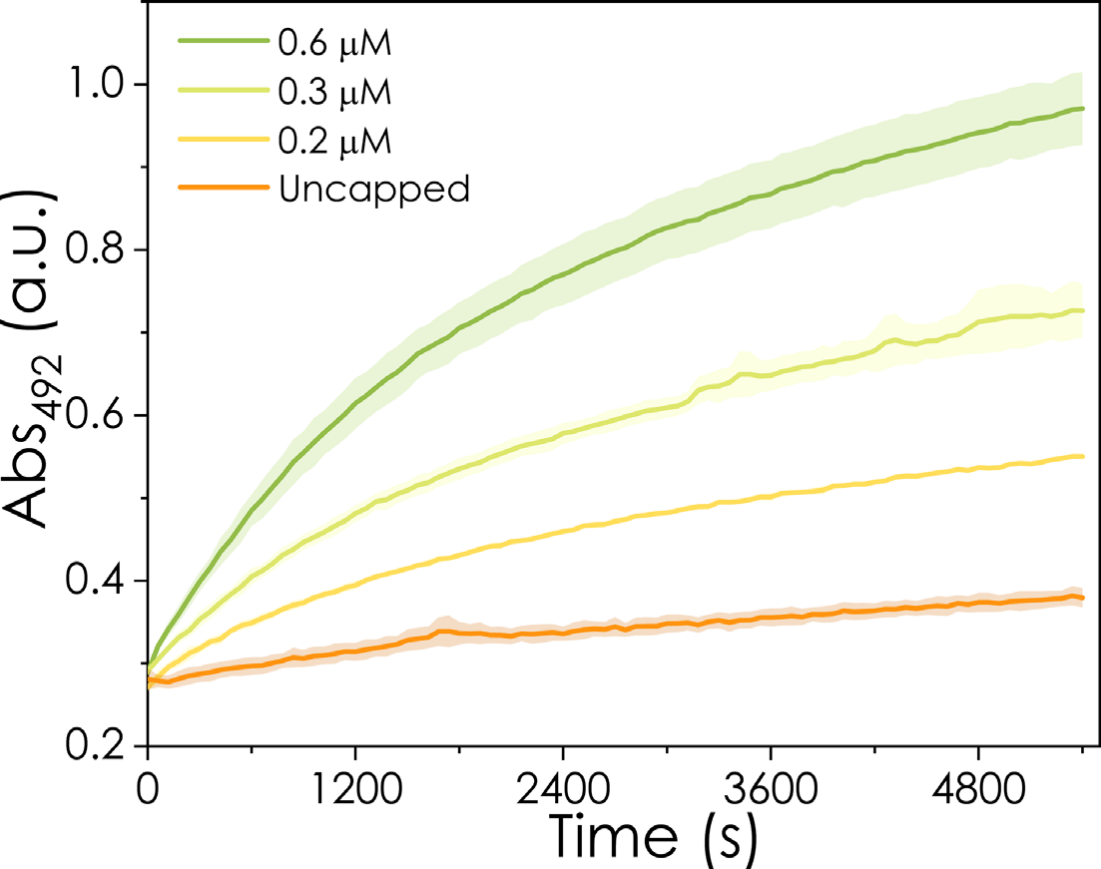
Biosensor response for a 6507 nt mRNA vaccine. Three different concentrations of the vaccine were tested to examine if the loss in sensitivity due length of the mRNA tested can be compensated for by increasing the concentration. In all cases 4 μg of beads were used for the colorimetric assay.

The results shown in Figure 6 indicate that the B4E biosensor remains functional when analysing longer mRNAs. This is the first example of a system able to simultaneously analyse the integrity and capping level of long and complex mRNAs such as those developed as vaccines. The biosensor was still able to distinguish between capped and noncapped mRNAv. However, a noticeable decrease in the dynamic range was observed when compared to the longest ARCA capped mRNA, cRNA_3_, likely due to less efficient binding of RNAs of increasing length (Supplementary Table S4). Nonetheless, this loss can be compensated for by increasing the amount of mRNA used for the assay. Despite requiring a larger amount of sample on a mass basis (e.g. for mRNAv at 0.6 μM, 25.13 μg were needed, as opposed to the 0.89 μg or 0.6 μM for cRNA_1_, or 5.03 μg or 0.3 μM for cRNA_3_), the amount is still in the range of a single dose of an mRNA-based vaccine and hence would still be feasible for a quality control assay (51,52). It should also be noted that the baseline signal corresponding to uncapped mRNAv is equivalent to those observed previously with the other uncapped controls (Figure 3). Hence, the overall noise in the assay is not intensified by longer mRNAs, probably due to a trade-off between the less efficient interaction between the beads and mRNAs (Supplementary Table S4), which limits both the non-specific binding and the dynamic range of the biosensor for longer chains.

Taken together, the results for mRNAv and those for the co-transcriptionally capped mRNAs with ARCA nucleotides show that the biosensor is active across a wide range of molecule sizes and different 5′-UTR and polyA tail environments with minimal adjustments. The biosensor showed activity for the two main capping approaches for *in vitro* transcribed mRNAs: ARCA and vaccinia virus capping enzyme. This could be relevant from both the lab-scale and manufacturing standpoint, facilitating rapid optimization of the capping conditions tailored to specific mRNA chains regardless of synthesis scale. In this sense, the impact of 5′-UTR on the capping efficiency of the vaccinia protein, which has been shown to vary, can be quickly screened with the B4E biosensor (53,54). Finally, a fully calibrated system able to monitor key points in the manufacturing process would reveal batch-to-batch variation in the synthesis or purification steps via the detection of aberrant signals.

##### *In vivo* transcribed mRNAs

To further explore the applicability of the biosensor, its performance with mRNA extracted from cells was tested to explore the viability of analysing mRNAs transcribed *in vivo*. The aim was to determine whether the biosensor can be used to characterise the quality of mRNA extracts (in terms of percentage of mRNA versus ribosomal RNA and/or fragmentation of transcripts), which is a key parameter for downstream applications, e.g. for RNA-seq or other applications. First, we tested the response of the biosensor with commercial tRNA from wheat germ and with total RNA extracted from CHO cells (which contains large amounts of ribosomal RNA), neither of which produced a significant response (Supplementary Figure S12).

**Figure 7.**
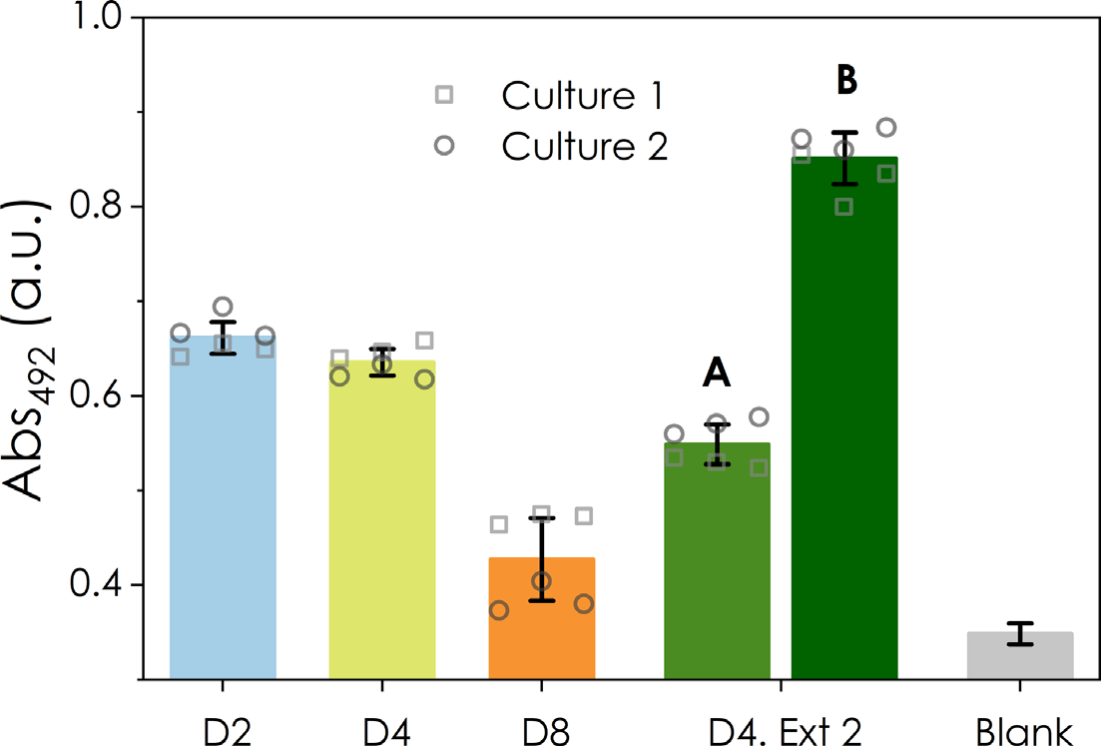
Biosensor response for mRNA extracted from cultured Chinese Hamster Ovary cells. D2, D4 and D8 correspond to the signals obtained for the mRNA extracted from 10^6^ cells at days 2, 4 and 8 of cell culture. A second extraction method (D4. Ext 2) was tested for day 4, using 2.33 × 10^6^ cells per extraction. For these samples, the amount of mRNA corresponding to 10^6^ and to 3.3 × 10^6^ cells was loaded to the sensor (columns A and B, respectively). The bars show the response after 3000 s. In this case, for the biosensor assay 50 μg of beads were used, keeping the rest of the volumes as indicated in materials and methods section. For the final colorimetric assay, 8 μg of beads were used.

Next, using a kit designed to isolate mRNA, we extracted mRNA from 1 × 10^6^ CHO cells on three different days of culture and loaded these onto the biosensor. Figure 7 shows the response after 50 min of the colorimetric assay. Both the ratio of the sample to beads and the amount of beads used in the nitrocefin assay had to be increased to improve the dynamic range. However, the B4E biosensor was able to detect differences in sample quality. For example, a clear signal is observed for days 2 and 4 of culture, when cells are in exponential growth phase, while for day 8, when the culture reached a late stationary phase and the cell viability dropped below 90%, almost no signal was detected. This response is in agreement with the amounts of RNA purified from each sample (Supplementary Table S5) and the expected purity of the mRNA extracts at the different culture stages studied. The biosensor can also be used as a tool to optimize mRNA extractions. For example, on day 4 of culture, extractions using 2.33 × 10^6^ cells was also done to explore the effect of cell number on mRNA quality. The signal of the biosensor slightly decreased when loading the mRNA amount corresponding to 1 × 10^6^ cells from this second purification (Figure 7, D4. Ext. 2 A column), suggesting lower yield of extraction per number of cells used and/or a bigger amount of contamination with ribosomal RNA. The biosensor is also able to detect different concentrations of extracted mRNAs, as shown when loading the mRNA extracted from 3.3 × 10^6^ cells (Figure 7, D4. Ext. 2 B column).

Finally, to test if the biosensor response was also specific to capped mRNAs *in vivo*, we treated mRNA extracts with the Cap-Clip™ acid pyrophosphatase enzyme to remove the cap structure. Decapping the mRNAs produced a decrease in the biosensor signal compared to the response for the control reaction without enzyme (Supplementary Figure S12). However, the sample treated with Cap-Clip™ still shows a signal above the background, possibly due to incomplete enzymatic digestion or interactions of the B4E enzyme with other features of the mRNA. For example, previous observations report that the presence of a *N*^6^-2’*O*-dimethyladenosine group (m^6^Am) adjacent to the m^7^-GTP cap confers reduced susceptibility to mRNA decapping by the enzyme DCP2, either *in vitro* and or *in vivo* (55). mRNA extracted from cells may have a high degree of variability in sequence and post-transcriptional modification, so secondary factors may lead to changes in biosensor performance. Depending on the research question of interest, further investigations could be undertaken to explore interactions of B4E with other post-transcriptional modifications. Overall, the results suggest the B4E biosensor is applicable to mRNAs transcribed *in vivo*, which can be of interest for research purposes, such as studying intracellular mRNA levels for example, or simply to analyse the quality of mRNA extracts and develop optimized extraction protocols.

In summary, in this work we present a biosensor able to simultaneously detect the integrity and presence of m^7^G structures on mRNA molecules. It has been designed to be compatible with low technological requirements and to bridge the current gap between easily accessible techniques for analysing RNA that provide very little information (e.g. enzymatic digestion, gel electrophoresis) and specialist techniques that are more difficult to perform, but provide more information (e.g. splinted ligation PCR, LC-MS). None of the current technologies offer this feature, and here we show that it is applicable for a wide range of RNA sizes. This was achieved by the construction of the chimeric protein B4E, composed of the murine m^7^G-binding protein eIF4E and the enzyme β-lactamase, thus combining the recognition and transduction functions required in a sensor. Functionalised resin, biolayer interferometry and m^7^GTP inhibition analyses demonstrate the specific nature of the interaction of B4E towards m^7^G structures, confirming that the functionality of eIF4E was preserved in the chimeric protein.

Remarkably, the B4E biosensor response is linear against the capping percentage of *in vitro* transcribed mRNAs and remained unaltered for mRNA sizes ranging from 250 to 2700 nt, without a noticeable decrease in the dynamic range. Therefore, due to the ubiquitous presence of cap structures on mRNAs, this system would be valid to analyse the capping level and integrity of a variety of mRNA samples in a single step with minimal adaptation for each new target molecule. The main advantage of the B4E biosensor lies in the fact that it does not require alteration of the mRNA sequence or further amplification reactions, as opposed to alternative cap detection options. It also allows simultaneous detection of the cap and polyA structures, which gives information about both capping level and integrity of the mRNA in a single measurement that is easy to perform.

The applicability of the system was further explored using more complex mRNA samples. In the first instance, capped and uncapped versions of an mRNA vaccine under investigation were assayed, generating similar responses to the previously tested *in vitro* transcribed mRNAs, and demonstrating the functionality with chains up to 6.5 kb. Furthermore, the B4E biosensor could be used to measure the amount of mRNA extracted CHO cells from different days of a batch culture, demonstrating its ability to analyse mRNA extracts from living eukaryotic cells.

We envisage that the next step to further expand the applicability of the BE4 biosensor could be enabling absolute quantification of capped and non-degraded mRNA concentrations. This could be achieved by a detailed characterization of the degradation kinetics of nitrocefin by BE4 along with an accurate measurement of the mRNA-protein interactions for example.

Overall, these results demonstrate the wide applicability of the B4E biosensor, spanning from simple visual inspections to more advances analyses suited for laboratories. To our knowledge, this is the first report on a biosensor enabling an on-site analysis of the integrity and capping status of mRNAs. Therefore, because of its simplicity, the B4E biosensor could support the manufacture and administration of healthcare products where mRNAs are the active component to a wider group of users. Simultaneously, it can be applied for research or industrial purposes such as *in vitro* syntheses of mRNAs, where it could facilitate the optimization of capping enzymes or reactions conditions, to study the level of degradation or aborted transcripts or for tracking the insertion of chemically modified caps to increase the expression of exogenously delivered mRNAs in therapeutics, for example (27,53). Additionally, as an advanced research application, the flexibility of the B4E biosensor enables the detection of cap structures on mRNA extracts from cells. This could serve as the basis for new research projects, since the availability of a biosensor designed for the study of cap structures offers considerable simplification of the detection mRNA capping compared to other existing methods. For example, a further evolution of the cap-recognition element, such as the ability to differentiate between cap 0 and cap 1 structures, would be of interest to elucidate still unknown roles of mRNA capping and methylation (25,53).

## Supplementary Material

gkaa955_Supplemental_File
