# Supplementary material for: High resolution biosensor to test the capping level and integrity of mRNAs
Source: Nucleic Acids Res. 2020 Nov 5;48(22):e129. doi: 10.1093/nar/gkaa955 (PMC7736790; doi:10.1093/nar/gkaa955)
Supplement: gkaa955_Supplemental_File [file gkaa955_supplemental_file.pdf]

SUPPLEMENTARY INFORMATION

High resolution biosensor to test the capping level and integrity of mRNAs

Ignacio Moya-Ramírez[1,2,*], Clement Bouton[3], Cleo Kontoravdi[1], Karen Polizzi[1,2,*]

[1] Department of Chemical Engineering, Imperial College London, London, SW7 2AZ, United Kingdom

[2] Imperial College Centre for Synthetic Biology, Imperial College London, London, SW7 2AZ, United Kingdom

[3] Department of Infectious Disease, Imperial College London, London, W2 1NY, United Kingdom

* To whom correspondence should be addressed: nachmoya@gmail.com; kpolizzi@ic.ac.uk

1. SUPPLEMENTARY METHODS

1.1. Plasmid construction for protein expression and purification

Plasmid pET28a+ (Novagen, UK) was used for protein expression. The synthetic gene encoding B4E was PCR amplified with primers 1 and 2 (Supplementary Table 1), the pET28a+ backbone was amplified with the primers 3 and 4 and both parts assembled via Gibson DNA assembly (40), to generate the plasmid pET28a-B4E-vi. A second version of the expression plasmid, pET28a-B4E-v.ii, was prepared by deleting some additional residues from N- and C-termini of B4E-v-i. Here primers 5 and 6 were used to amplify B4E from the original gene synthesis vector and primers 7 and 8 to amplify the pET28a+ plasmid backbone. Both fragments were joined using Gibson assembly. For the construction of the plasmid pET28a-βLac the *ampR* gene was amplified with primers 9 and 10 from the pET-15b plasmid and the pET28a+ backbone was amplified with primers 11 and 12. The fragments were joined using Gibson DNA assembly.

1.2. SDS-PAGE analysis of B4E

B4E was expressed and purified as described in the main manuscript. Figure S1 shows, the SDS-PAGE analysis of the various fractions obtained from his-tag purification. Elutions 2, 3 and 4 (lanes 8-10) were combined and buffer exchanged using a centrifugal concentrator with a 30 kDa MWCO (Vivaspin). 200 µL of purified B4E at 663 µg/mL were obtained (as measured by absorbance at 280 nm).

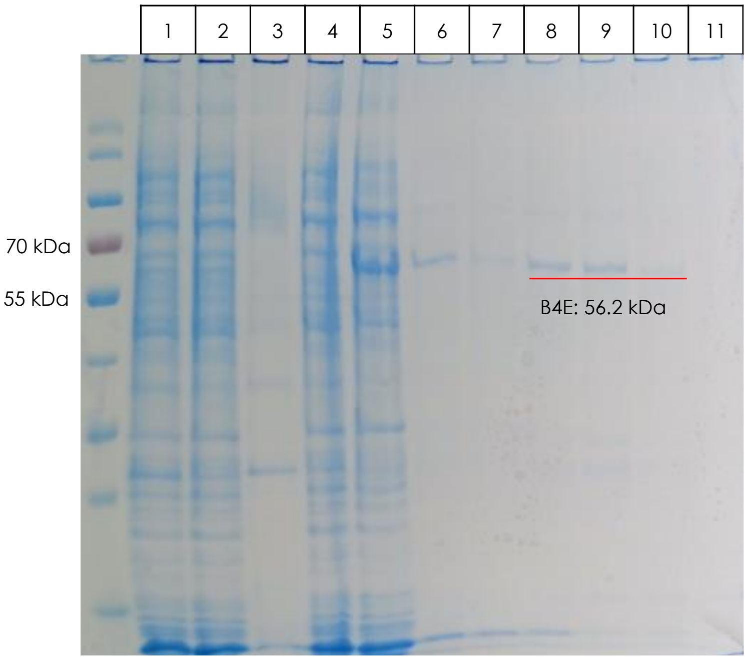

Figure S1. 10 % SDS- polyacrylamide gel showing the different fractions for the purification of B4E protein, PageRuler (Thermo) is used as a molecular weight marker. Lane 1: cell lysate; lane 2: soluble fraction; lane 3: insoluble fraction; lanes 4: flow through (fraction not bound to the resin); lanes 5 and 6: washes; lanes 7 to 11: elutions 1 to 5. The expected size of the B4E protein is 56.2 kDa.

1.2. Purification of *in vitro* transcribed (IVT) RNAs

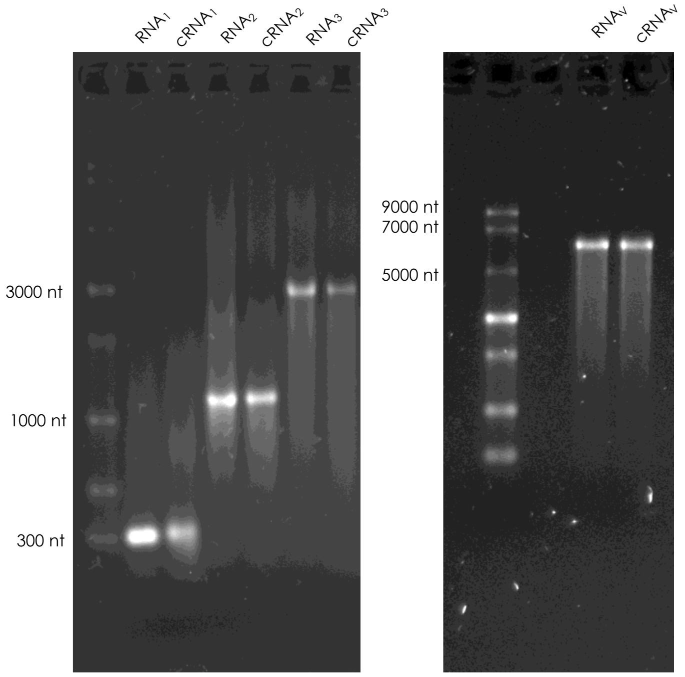

Figure S2. 1.5 % agarose gel showing the different IVT RNAs used in this study (~600 ng of sample loaded).

Table S1. Yields and purity of the IVT RNAs used in this study.

	Concentration (ng/µL)	Yield (µg)	260/230	260/280
RNA_1	857.6	30	2.831	2.393
$cRNA_1$	820	28.7	2.228	2.032
RNA_2	1047.2	36.22	2.012	1.838
$cRNA_2$	632.8	22.15	2.782	2.401
RNA_3	550.2	35.22	2.454	2.209
$cRNA_3$	781.6	25.01	2.49	2.186
RNA_v	3100	-	2.194	2.137
$cRNA_v$	3100	-	2.471	2.298

1.3. Decapping reaction of mRNA from CHO cells

An aliquot of eukaryotic mRNA (~ 800 ng) extracted from 10^6 CHO cells following the method described in the main text, was digested with 7.5 U of Cap-Clip™ acid phosphatase (CellScript, US) in a 50 µL reaction volume at 37 ºC for three hours. Prior to the addition of the enzyme and reaction buffer, the mRNA sample was heated at 60 ºC for 10 min and then snap cooled in an ice bath. The decapped sample was purified and concentrated using the RNA Clean & Concentrator kit (Zymo Research).

1.4. Total RNA extraction from CHO cells for biosensor control experiments

The total RNA fromCHO cells was extracted from 10^6 CHO cells collected on day 4 of culture produced as indicated in the materials and methods section. The RNA was purified from 0.5 mL of cell lysate with a RiboPure™ RNA purification kit (Thermo), with the addition of the Phenol:Chloroform:IAA step (omitting the use of zirconia beads) and continuing with the purification as indicated by the manufacturer.

1.5. Capping efficiency of IVT mRNAs

To estimate the percentage of capped RNAs incorporated either co-transcriptionally for $cRNA_1$, $cRNA_2$ and $cRNA_3$ or post-transcriptionally for $mRNA_v$, 5 µg of RNA was first digested with an RNA 5' polyphosphatase (Lucigen, UK), leaving 5' monophosphorylated chains on the uncapped molecules of the RNA sample. Reactions were performed in a 20 µL final volume, at 37ºC for 45 min. The digested samples were purified with the RNA Clean & Concentrator kit (Zymo Research). Subsequently, 500 ng of the purified RNA was digested with Terminator™ 5' to 3' ribonuclease (Lucigen), to degrade the 5' monophosporylated RNAs. Reaction were performed in a 20 µL final volume at 30ºC for 30 min for $cRNA_1$ and for 15 min for the other RNAs. Reaction were stopped by mixing with an equal volume of 2x RNA loading dye (NEB) in an ice bath. Samples were run on a 1.5 % agarose gel in TBE buffer at 110V (Figure S3). The percentage of capped RNA was calculated by densitometry by comparison with a control reaction lacking the ribonuclease using the image analysis software Fiji 2.

2. SUPPLEMENTARY RESULTS

2.1. Biosensor optimization

Several alternative assay conditions were studied to decrease non-specific binding between B4E and RNA. The pH was increased in order to decrease positive charges on the surface of the protein. However, increasing pH was not effective, since even though the non-specific binding was decreased, the B4E binding to capped RNA was also drastically reduced (Figure S8). The optimal pH for the interaction between eIF4e and m^7GTP is approximately 7.25, and is influenced by zwitterionic nature of the m^7GTP group, which binds in its cationic form to eIF4e (1).

Alternatively, altering the reducing conditions in the buffer was a more effective way to reduce non-specific binding. Figure S9 shows the response of the biosensor with and without addition of DTT to buffer B. Adding DTT considerably reduces non-specific binding, likely by preventing intermolecular B4E oligomerisation via disulfide bond formation (2) and electrostatic interaction between the positively charged region of the oxidised cysteines of the B4E and the negatively charged mRNA, pdT_{25} oligo and beads. This observation is also in agreement with the fact that eIF4E is naturally present in the cell nucleus and cytoplasm, which are reducing environments. Finally, further reduction of non-specific binding to an almost complete reduction of the background signal was achieved by the removal of non-functional amino acids on the C and N termini of the B4E (Figure S10).

2.2. Biosensor m^7GTP inhibition analysis

A biosensor assay using a buffer with a low level of stringency to permit non-specific binding further confirmed the specificity of the interaction between B4E and the mRNA cap during the actual biosensor assay. The presence of the m^7GTP is expected only to affect the B4E-cap 0 interaction, since some of the eIF4e peptides will bind the methylated nucleotide instead of capped RNA, while the controls should remain unaffected. This was confirmed experimentally, as shown in Figure S11. The presence of m^7GTP leads to a decrease in the signal of the biosensor when $cRNA_1$ is used, while the signal due to non-specific binding observed for RNA_1 or the pdT_{25} oligo-functionalised beads was unchanged. This further confirms the existence of a specific interaction between the cap binding region of B4E and the cap 0 of mRNA while immobilised on the beads used for the biosensor assay. In addition, this experiment suggests that the background signal originates from non-specific interactions with other regions of the protein.

3. OLIGOS AND SEQUENCES

Table S2. Oligos used for PCR amplification and latter Gibson assembly.

Nº-Orientation	Target	Sequence
1-Fwd	pRSET-B4E. Amplification of B4E gene.	CTTTAAGAAGGAGATATACCATGCGGGGTTCTCATCATCA

2-Rev		TTGTTAGCAGCCGGATCTCACTACTCGAGAAC AACAAAACG
3-Fwd	pET28a+ backbone amplification	GTTTTGTTGTTCTCGAGTAGTGAGATCCGGCT GCTAACAA
4-Rev		TGATGATGAGAACCCCGCATGGTATATCTCCT TCTTAAAGTTAAACA
5-Fwd	pRSET-B4E. Amplification of B4E gene.	GGAGATATACCAATGCACCACCACCACCACC ACATGCATCCGGAAACACTGGTT
6-Rev		TTGTTAGCAGCCGGATCTCAAACAACAAAACG ATTTTTGGTGG
7-Fwd	pET28a+ backbone amplification	CCAAAAATCGTTTTGTTGTTTGAGATCCGGCT GCTAACAA
8-Rev		CGGATGCATGTGGTGGTGGTGGTGGTGCATT GGTATATCTCCTTCTTAAAGTTAAAC
9-Fwd	pET15b. Amplification of AmpR gene	CAGTGGTGGTGGTGGTGGTGCCAAT GCTTAATCAGTGAGG
10-Rev		AAGAAGGAGATATACCAATGCACCC AGAAACGCTGGTGA
11-Fwd	pET28a+ backbone amplification	CCTCACTGATTAAGCATTGGCACCA CCACCACCACCACTG
12-Rev		ACCAGCGTTTCTGGGTGCATTGGTATATCTCC TTCTTAAAGTTAAACAAA ATTATTT

- DNA sequence of BAE-v.i,. Red font indicates the the nucleotides removed to generate B4E-v.ii:

ATGGCTAGCATGACTGGATCCATGCATCCGGAAACACTGGTTAAAGTTAAAGATGCCGAAGATCA
GCTGGGTGCACGTGTTGGTTATATTGAACTGGATCTGAATAGCGGCAAAATCCTGGAAAGTTTTC
GTCCGGAAGAACGCTTTCCGATGATGAGCACCTTTAAAGTTCTGCTGTGTGGTGCAGTTCTGAG
CCGTGTTGATGCAGGTCAAGAACAGCTGGGACGTCGTATTCATTATAGCCAGAATGATCTGGTT
GAATATAGTCCGGTTACCGAAAAACATCTGACCGATGGTATGACCGTTCGTGAACTGTGTAGCG

CAGCAATTACCATGAGCGATAATACCGCAGCAAATCTGCTGCTGACCACCATTGGCGGTCCGAA
AGAACTGACCGCATTTCTGCATAATATGGGTGATCATGTTACCCGTCTGGATCGTTGGGAACCTG
AACTGAATGAAGCAATTCCGAATGATGAACGTGATACCACCATGCCTGCAGCAATGGCAACCAC
ACTGCGTAAACTGCTGACCGGTGAACTGTTAACCCTGGCAAGCCGTCAGCAGCTGATCGATTGG
ATGGAAGCAGATAAAGTTGCAGGTCCGCTGCTGCGTAGCGCACTGCCTGCAGGTTGGTTTATTG
CAGATAAAAGCGGTGCCGGTGAACGTGGTAGCCGTGGTATTATTGCCGCACTGGGTCCTGATG
GTAAACCGAGCCGTATTGTTGTGATTTATACCACCGGTAGCCAGGCAACCATGGATGAACGTAAT
CGTCAGATTGCAGAAATTGGTGCAAGCCTGATTAAACATTGGGGTGCTGGTGCCGGTGCGGGTG
CCGGTGCTGGTGCTGGTGCTGGTGCCGGTGCAGGCGCAATGGCAACCGTTGAACCGGAAACCA
CACCGACCACCAATCCGCCTCCGGCAGAAGAAGAAAAAACCGAAAGCAATCAAGAAGTTGCCAA
TCCGGAACACTATATCAAACATCCGCTGCAGAATCGTTGGGCACTGTGGTTTTTCAAAAACGACA
AAAGCAAAACCTGGCAGGCCAATCTGCGTCTGATTAGCAAATTTGATACCGTGGAAGATTTCTGG
GCTCTGTATAATCATATTCAGCTGAGCAGCAATCTGATGCCTGGTTGTGATTATAGCCTGTTTAAA
GATGGTATTGAGCCGATGTGGGAAGATGAAAAAAACAAACGTGGTGGTCGTTGGCTGATTACCC
TGAATGCACAGCAGCGTCGTAGCGATCTGGATCGCTTTTGGCTGGAAACCCTGCTGTGTCTGAT
TGGTGAAAGTTTCGATGATTATTCGGATGATGTTTGTGGTGCCGTTGTTAATGTTCGTGCAAAAG
GTGATAAAATTGCCATTTGGACCACCGAATGTGAAAATCGTGATGCAGTTACCCATATTGGTCGC
GTTTATAAAGAACGTCTGGGTCTGCCTCCGAAAATTGTTATTGGTTATCAGAGCCATGCAGATAC
CGCAACCAAAAGCGGTAGCACCACCAAAAATCGTTTTGTTGTTCTCGAG*

The following are the sequences of the mRNAs transcribed *in vitro* and tested with the biosensor.

- $mRNA_1$

GGGGAAUUGUGAGCGGAUAACAAUUCCCCUCUAGAAAUAAUUUUGUUUAACUUUAAGAAGGA
GAUAUACCAUGGGCAGCAGCCAUCAUCAUCAUCAUCACAGCAGCGGCCUGGUGCCGCGCGGC
AGCCAUAUGGCUAGCAUGACUGGUGGACAGCAAAUGGGUCGCGGAUCCGAAUUCGAGCUCCG
UCGACAAGCUUGCGGCCGCAAAAAAAAAAAAAAAAAAAAAAAAAAAAAAAAAAAAAAAAAAAAAA
AAAA

- $mRNA_2$

GGGGAAUUGUGAGCGGAUAACAAUUCCCCUCUAGAAAUAAUUUUGUUUAACUUUAAGAAGGA
GAUAUACCAUGGGCAGCAGCCAUCAUCAUCAUCAUCACAGCAGCGGCCUGGUGCCGCGCGGC
AGCCAUAUGGCUAGCAUGACUGGUGGACAGCAAGCGGGAUAUAACAUGAGCUGUCUUCGGUA
UCGUCGUAUCCCACUACCGAGAUAUCCGCACCAACGCGCAGCCCGGACUCGGUAAUGGCGCG
CAUUGCGCCCAGCGCCAUCUGAUCGUUGGCAACCAGCAUCGCAGUGGGAACGAUGCCCUCAU
UCAGCAUUUGCAUGGUUUGUUGAAAACCGGACAUGGCACUCCAGUCGCCUUCCCGUUCCGCU
AUCGGCUGAAUUUGAUUGCGAGUGAGAUAUUUAUGCCAGCCAGCCAGACGCAGACGCGCCGA
GACAGAACUUAAUGGGCCCGCUAACAGCGCGAUUUGCUGGUGACCCAAUGCGACCAGAUGCU
CCACGCCCAGUCGCGUACCGUCUUCAUGGGAGAAAAUAAUACUGUUGAUGGGUGUCUGGUCA
GAGACAUCAAGAAAUAACGCCGGAACAUUAGUGCAGGCAGCUUCCACAGCAAUGGCAUCCUG

GUCAUCCAGCGGAUAGUUAAUGAUCAGCCCACUGACGCGUUGCGCGAGAAGAUUGUGCACCG
CCGCUUUACAGGCUUCGACGCCGCUUCGUUCUACCAUCGACACCACCACGCUGGCACCCAGU
UGAUCGGCGCGAGAUUUAAUCGCCGCGACAAUUUGCGACGGCGCGUGCAGGGCCAGACUGG
AGGUGGCAACGCCAAUCAGCAACGACUGUUUGCCCGCCAGUUGUUGUGCCACGCGGUUGGG
AAUGUAAUUCAGCUCCGCCAUCGCCGCUUCCACUUUUUCCCGCGUUUUCGCAGAAACGUGGC
UGGCCUGGUUCACCACGCGGGAAACGGUCUGAUAAGAGACACCGUUCGAGCUCCGUCGACAA
GCUUGCGGCCGCAAAAAAAAAAAAAAAAAAAAAAAAAAAAAAAAAAAAAAAAAAAAAAAA

- mRNA3

GGGGAAUUGUGAGCGGAUAACAAUUCCCCUCUAGAGCGCCGAACUGGGCCCUGAAGAAGGGC
CCCAUAUGGCUAGCAUGACUGGUGGACAGCAACCGUUUAGGUGUUUUCACGAGCAAUUGACC
AACAAGGACCAUAGAUUAUGAAAAUCGAAGAAGGUAAACUGGUAAUCUGGAUUAACGGCGAUA
AAGGCUAUAACGGUCUCGCUGAAGUCGGUAAGAAAUUCGAGAAAGAUACCGGAAUUAAAGUCA
CCGUUGAGCAUCCGGAUAAACUGGAAGAGAAAUUCCCACAGGUUGCGGCAACUGGCGAUGGC
CCUGACAUUAUCUUCUGGGCACACGACCGCUUUGGUGGCUACGCUCAAUCUGGCCUGUUGG
CUGAAAUCACCCCGGACAAAGCGUUCCAGGACAAGCUGUAUCCGUUUACCUGGGAUGCCGUA
CGUUACAACGGCAAGCUGAUUGCUUACCCGAUCGCUGUUGAAGCGUUAUCGCUGAUUUAUAA
CAAAGAUCUGCUGCCGAACCCGCCAAAAACCUGGGAAGAGAUCCCGGCGCUGGAUAAAGAAC
UGAAAGCGAAAGGUAAGAGCGCGCUGAUGUUCAACCUGCAAGAACCGUACUUCACCUGGCCG
CUGAUUGCUGCUGACGGGGGUUAUGCGUUCAAGUAUGAAAACGGCAAGUACGACAUUAAAGA
CGUGGGCGUGGAUAACGCUGGCGCGAAAGCGGGUCUGACCUUCCUGGUUGACCUGAUUAAA
AACAAACACAUGAAUGCAGACACCGAUUACUCCAUCGCAGAAGCUGCCUUUAAUAAAGGCGAA
ACAGCGAUGACCAUCAACGGCCCGUGGGCAUGGUCCAACAUCGACACCAGCAAAGUGAAUUA
UGGUGUAACGGUACUGCCGACCUUCAAGGGUCAACCAUCCAAACCGUUCGUUGGCGUGCUGA
GCGCAGGUAUUAACGCCGCCAGUCCGAACAAAGAGCUGGCAAAAGAGUUCCUCGAAAACUAU
CUGCUGACUGAUGAAGGUCUGGAAGCGGUUAAUAAAGACAAACCGCUGGGUGCCGUAGCGCU
GAAGUCUUACGAGGAAGAGUUGGUGAAAGAUCCGCGUAUUGCCGCCACUAUGGAAAACGCCC
AGAAAGGUGAAAUCAUGCCGAACAUCCCGCAGAUGUCCGCUUUCUGGUAUGCCGUGCGUACU
GCGGUGAUCAACGCCGCCAGCGGUCGUCAGACUGUCGAUGAAGCCCUGAAAGACGCGCAGAC
UAAUUCGAGCUCGAACAACAACAACAAUAACAAUAACAACAACCUCGGGAUCGAGGGAAGGAU
UUCACAUAUGGCGACGCAAUCGGAAUAUGCGGAUCGCUUGGCAGCGGCGAUUGAAGCCGAAA
ACCAUUGCACGUCACAGACCCGCUUACUGAUCGAUCAAAUCAGUCAGCAACAAGGACGCAUUG
UAGCCCUGGAGGAACAGAUGAAACGUCAGGAUCAGGAGUGUCGCCAAUUACGUGCUUUGGUU
CAGGAUCUUGAGUCGAAAGGGAUUAAGAAAUUGAUCGGCAAUGUUCAAAUGCCUGUUGCUGC
AGUAGUGGUCAUGGCCUGCAAUCGUGCGGAUUACCUCGAGAAAACCAUCAAAUCCAUCCUGA
AGUAUCAGAUUAGCGUUGCACCGAAAUACCCUCUGUUUAUCUCCCAAGAUGGUUCUCAUCCG
GAUGUCCGCAAACUGGCGUUAAGCUACGAUCAACUGACCUAUAUGCAGCAUCUGGAUUUUGA
ACCGGUGCACACUGAACGUCCUGGCGAAUUAAUCGCGUAUUACAAAAUUGCACGCCACUACAA
AUGGGCCCUUGACCAGCUCUUUUACAAGCACAACUUUAGCCGGGUGAUCAUUCUUGAGGACG
AUAUGGAAAUUGCCCCAGACUUCUUCGACUUCUUUGAAGCCGGAGCUACUCUGCUGGAUCGC
GAUAAGUCGAUUAUGGCGAUCAGUAGCUGGAACGAUAACGGGCAGAUGCAGUUUGUGCAAGA

UCCCUAUGCUUUAUAUCGCUCAGACUUCUUUCCGGGUCUGGGUUGGAUGUUGAGUAAAUCGA
CAUGGGACGAACUGAGCCCGAAAUGGCCGAAAGCUUACUGGGAUGACUGGUUGCGCCUGAAG
GAAAACCAUCGUGGUCGUCAGUUCAUUCGCCCGGAAGUGUGUCGUAGCUAUAACUUUGGUGA
ACAUGGUAGCAGUCUGGGCCAGUUCUUUAAACAGUAUCUGGAACCCAUCAAACUCAAUGACG
UCCAGGUCGACUGGAAAUCCAUGGAUCUUUCUUAUCUGCUGGAGGACAAUUACGUGAAACAC
UUUGGCGAUCUGGUGAAGAAAGCGAAACCGAUUCAUGGUGCCGACGCAGUGCUGAAAGCGUU
UAACAUUGAUGGGGAUGUUCGCAUUCAGUACCGUGAUCAGCUGGACUUUGAAGAUAUUGCAC
GUCAGUUUGGCAUUUUCGAAGAGUGGAAAGAUGGCGUACCACGUGCGGCCUAUAAAGGCAUC
GUAGUGUUCCGCUAUCAGACGUCACGCCGGGUUUUCCUCGUCGGCCCAGACUCUCUGCAGC
AACUGGGCAAUGAAGAUACCGAAUUCGGUUCUGGUCUUAAUGAUAUUUUUGAAGCUCAGAAG
AUUGAAUGGCAUGUUCGAGCUCCGUCGACAAGCUUGCGGCCGCAAAAAAAAAAAAAAAAAAAA
AAAAAAAAAAAAAAAAAAAAAAAAAAAAAA

- 5’-UTR of mRNAv:

TTAAAACAGCTGTGGGTTGTCACCCACCCACAGGGTCCACTGGGCGCTAGCACTCTGGTACCTC
GGTACCTTTGTGCGCCTGTTTTATACCCCCCCCCCAGTTTGAAACTTAGAAGCAGCGCAAACCAG
ATCAATAGCAGGCATAACGCTCCAGTTATGTCTTGATCAAGCACTTCTGTTTCCCCGGACTGAGT
ATCAAT

- $mRNA_{1B}$

GGGGAAUUGUGAGCGGAUAACAAUUCCCCUCUAGAGCGCCGAACUGGGCCCUGAAGAAGGGC
CCCAUAUGGCUAGCAUGACUGGUGGACAGCAAAUGGGUCGCGGAUCCGAAUUCGAGCUCCGU
CGACAAGCUUGCGGCCGCAAAAAAAAAAAAAAAAAAAAAAAAAAAAAAAAAAAAAAAAAAAAAAA
AAA

- $cRNA_{\Delta pA}$

GGGGAAUUGUGAGCGGAUAACAAUUCCCCUCUAGAAAUAAUUUUGUUUAACUUUAAGAAGGA
GAUAUACCAUGGGCAGCAGCCAUCAUCAUCAUCAUCACAGCAGCGGCCUGGUGCCGCGCGGC
AGCCAUAUGGCUAGCAUGACUGGUGGACAGCAAAUGGGUCGCGGAUCCGAAUUCGAGCUCCG
UCGACAAGCUUGCGGCCGCAC

4. SUPPLEMENTARY TABLES AND FIGURES

Table S3. Parameters of the lines of best fit for the transfer functions displayed in Figure 4.

	Intercept	Slope (x10^3)	R^2
cRNA1	0.253	3.78	0.994
cRNA2	0.273	3.73	0.989
cRNA3	0.246	3.42	0.984

Table S4. Amounts of RNAs bound to the beads during the first incubation step of the biosensor assay. RNA concentrations of the samples before and after binding to the beads were measured on a BioDrop DUO+ microspectrophotometer. The percentage of the bound mRNA that is then bound to a E4B molecule was estimated using a calibration curve of the activity of the free protein. The calibration curve is shown in Figure S4.

	Initial concentration (µM)	Mass bound (ng)	pmol bound	% bound	% coverage with E4B
$cRNA_1$	0.6	346.67 ± 51.43	4.69 ± 0.69	68.42 ± 10.15	20.65
$cRNA_2$	0.6	853.3 ± 68.97	2.60 ± 0.21	20.70 ± 1.67	37.25
$cRNA_3$	0.3	960 ± 42.23	1.10 ± 0.04	16.50 ± 0.72	57.74
$cRNA_v$	0.3	1254.67 ± 108.24	0.60 ± 0.05	11.11 ± 0.96	75.85

Table S5. Amounts of mRNA (ng/µL) extracted from 10^6 CHO-S cells, for different days of cell culture.

	**Culture 1**	**Culture 2**
Day 2	41.6	76.8
Day 4	40.0	42.4
Day 4 (extraction 2)	53.2	41.7
Day 8	3.2	3.4

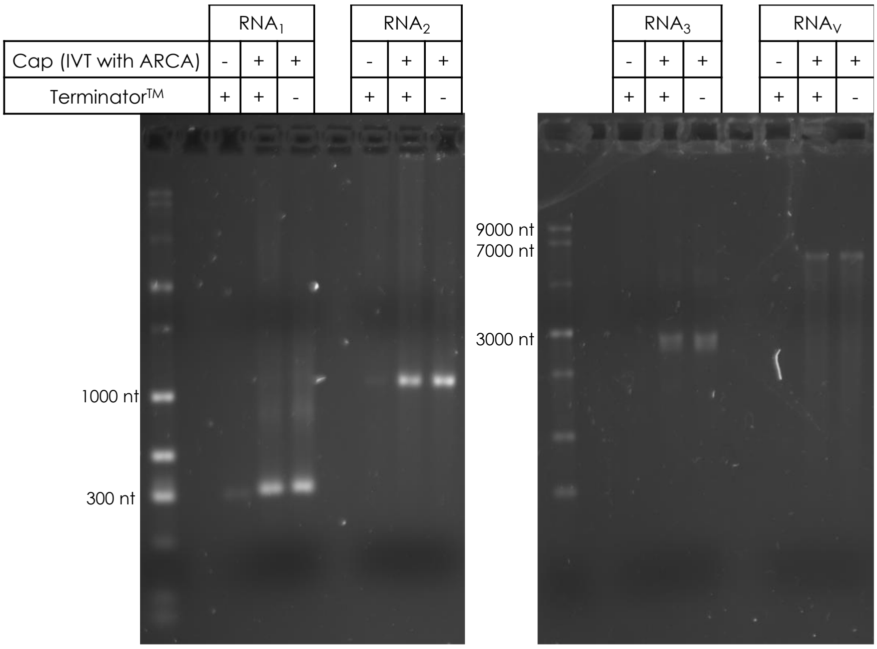

Figure S3. Estimation of the capped fractions of the mRNAs used in this study. Products of the 5' to 3' ribonuclease digestion of co-transcriptionally capped RNAs (RNA_1, RNA_2 and RNA_3) and post-transcriptionally capped RNA_v. The fractions of capped mRNA estimated by densitometry analyses comparing the digested samples with the reference ones are: $cRNA_1$ 93.3%, $cRNA_2$ 85.9%, $cRNA_3$ 82.5 % and $cRNA_v$ 82.6%

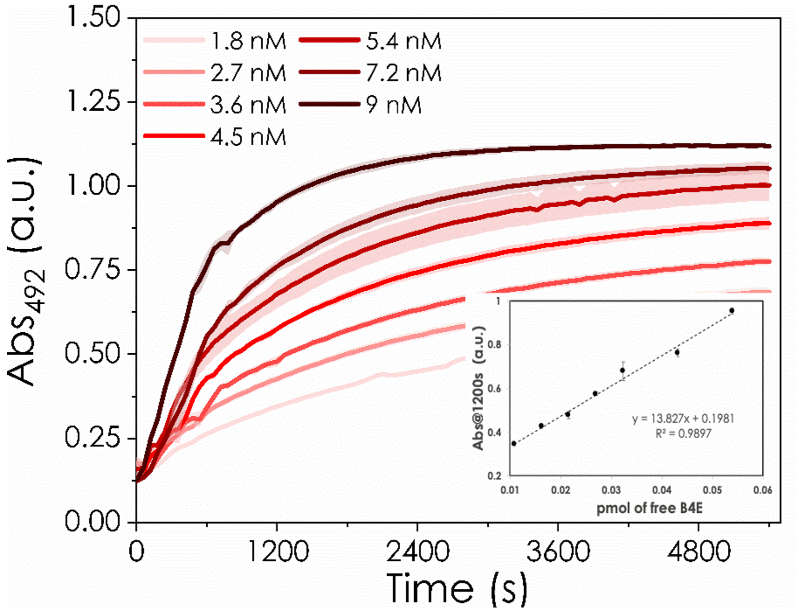

Figure S4. Calibration curve for free B4E. Spectroscopic measurement of nitrocefin hydrolysis at different concentrations of free B4E. The calibration curve (insert) is built by plotting the response at 1200 s versus the amount of free protein in the reaction.

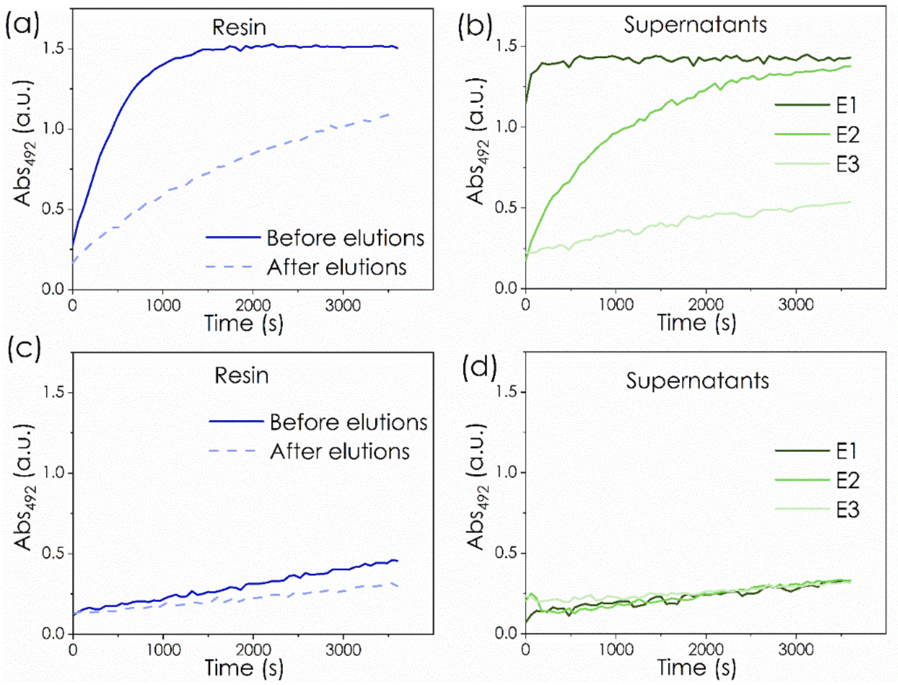

Figure S5. Biosensor assay with m^{7}GTP-functionalised resin loaded with B4E (a) and β-lactamase (c) and the elution fractions after addition of m^{7}GTP (b and d, respectively). Protein was bound to the resin and washed four times with buffer B (solid lines on "a" and "c", labelled as "Before elutions"). Subsequently, three elutions were performed using m^{7}GTP as the elution agent. Dashed lines in "a" and "c" correspond to assays performed with the resin post-elution. Figures "b" and "d" correspond to assays performed on the elution fraction. For the colorimetric assay, 5 µL of each elution fraction or 5 µL of resin resuspended in 100 µL of buffer B were used. The presence of protein (either bound to the resin or free in the elution fractions) can be detected by the hydrolysis of nitrocefin and the consequent increase in absorbance at 492 nm.

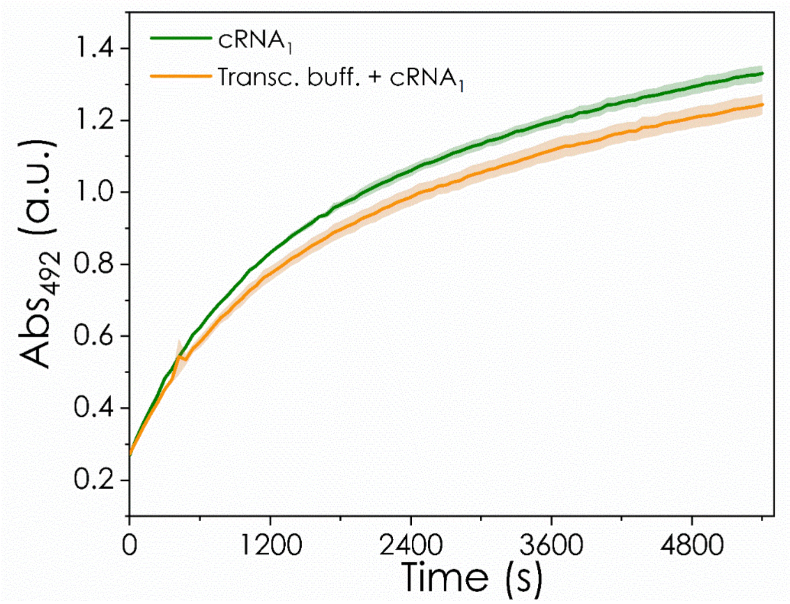

Figure S6. Biosensor response for capped mRNA ($cRNA_1$) in the presence some of the components of the T7 RNA polymerase reaction. $cRNA_1$ at 0.6 µM was mixed with 1 µg/µL linearized DNA template and 1.5 µM of each NTP to simulate a reaction before purification and then mixed with the beads to perform the biosensor assay.

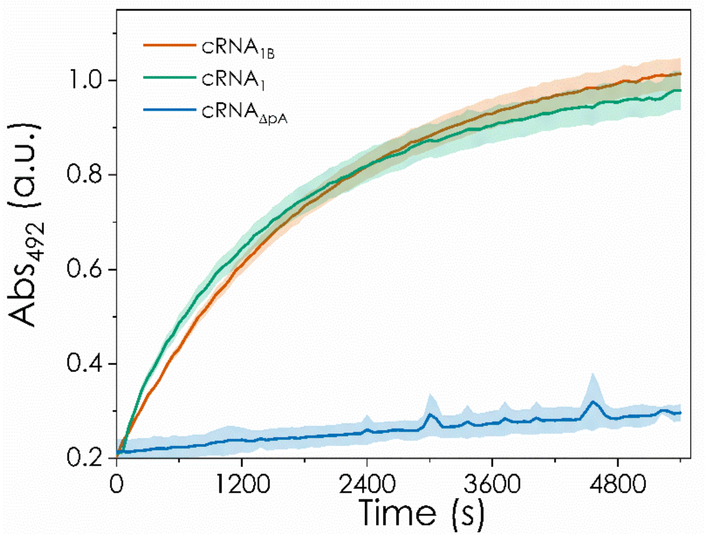

Figure S7. Biosensor assays analysing the effect of the presence of a boxB RNA hairpin in the 5' region of $cRNA_1$ ($cRNA_{1B}$) and the absence of polyA tail on the mRNA ($cRNA_{\Delta pA}$). Responses are compared to that obtained for standard $cRNA_1$. The RNA hairpin does not show a detrimental effect on the signal of the biosensor, while the absence of the polyA tail abolishes the signal, as expected from its inability to bind to the oligo dT-functionalised beads.

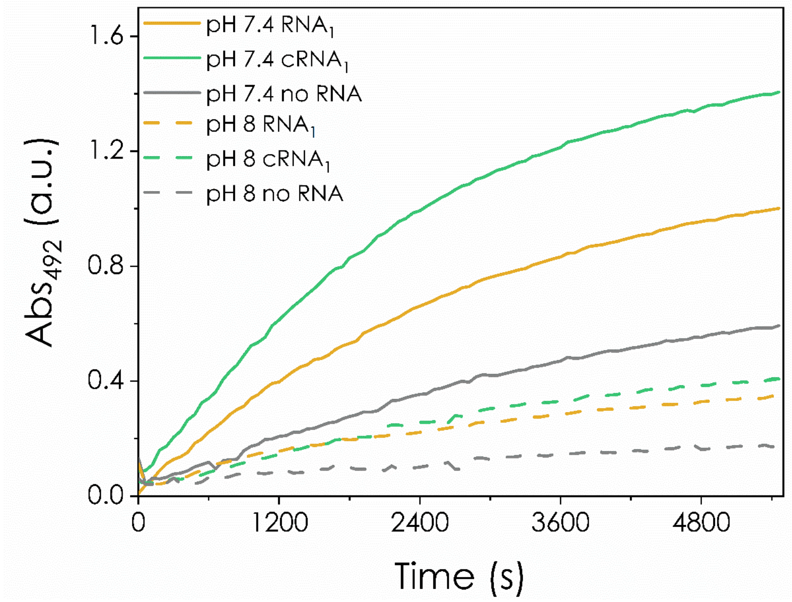

Figure S8. Biosensor assay for RNA_1 analysing the effect of the pH. The response of the sensor using buffer B at pH 7.4 (solid line) and pH 8 (dashed line). The background signal was almost eliminated at pH 8; However, the increase in pH also heavily reduces the specific interaction between B4E and $cRNA_1$.

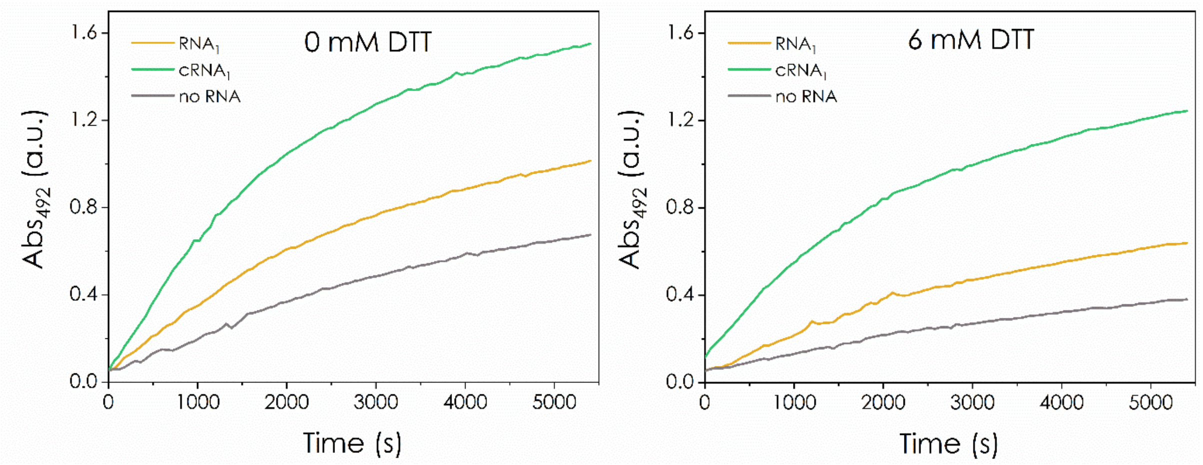

Figure S9. Biosensor assay analysing the effect of the addition of the reducing agent DTT to buffer B. DTT decreases the non-specific binding between B4E and uncapped RNA and beads. The signal for capped RNA was also reduced, likely due to screening out the non-specific interaction in this case as well.

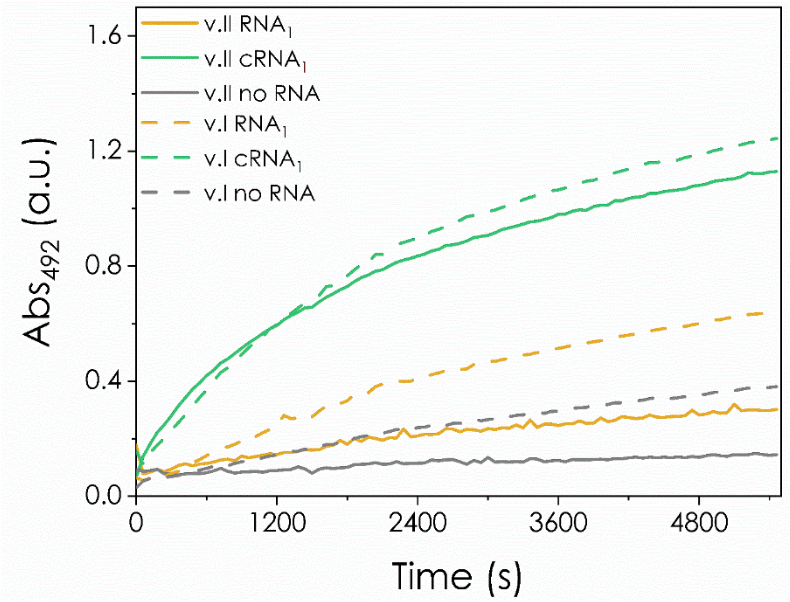

Figure S10. Biosensor assays comparing versions I and II of the protein (solid and dashed lines, respectively, see supplementary tables for information about the sequence). Standard conditions (those in Figure 3) were used. Both versions of the protein showed a similar affinity towards capped $mRNA_1$, while the non-specific binding was significantly reduced for version II.

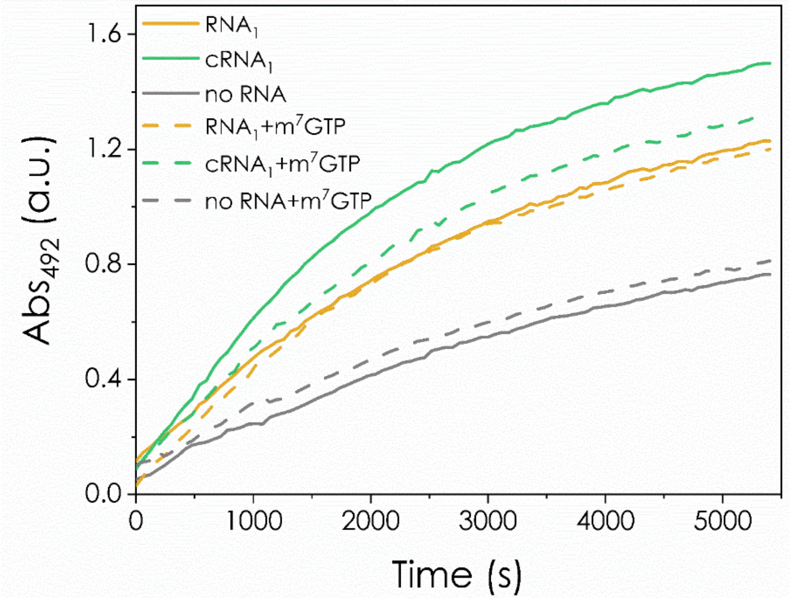

Figure S11. Biosensor assay for RNA_1 using buffer B' (HEPES 33 mM, KCl 66 mM, 0.1 % BSA, 0.1 % Tween20, pH 7.4). Buffer B' does not include DTT, therefore the non-specific binding between B4E and RNA and beads is higher. Higher non-specific binding allows the observation of the effect of the addition of 10 µM m^7GTP to the buffer (dashed line) compared to Buffer B' lacking m^7GTP (solid line). Free m^7GTP only reduces the signal for the capped RNA ($cRNA_1$), highlighting the non-specific nature of the interaction between B4E and uncapped RNAs and beads in buffers lacking DTT.

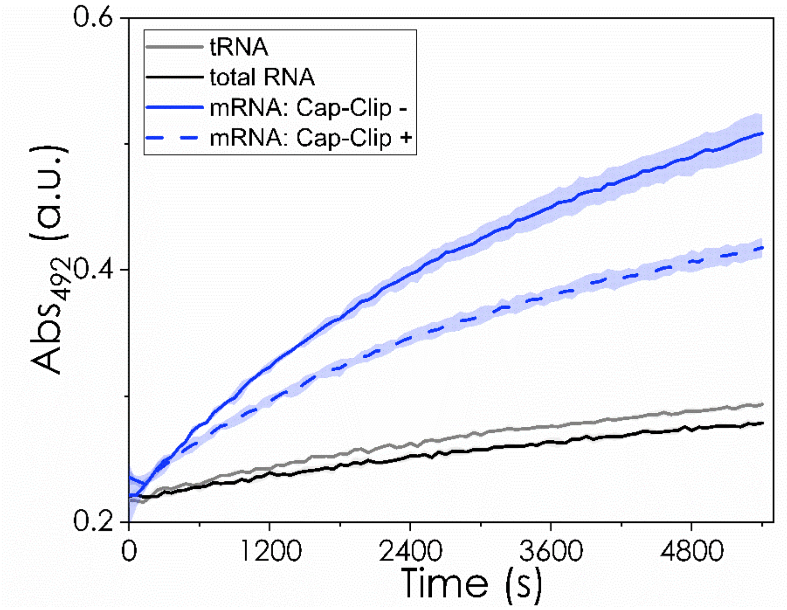

Figure S12. Biosensor assays of different RNAs produced *in vivo* . mRNA extracted from 10^6 CHO cells collected at the day 4 of culture treated with the decapping enzyme Cap-Clip acid phosphatase (blue dashed line) and a mock reaction without the addition of the enzyme (blue straight line). The biosensor was also tested with 50 ng/uL samples of tRNA (grey) total RNA extracted from CHO cells underthe same conditions (black). Averages and standard deviations of three experimental replicates are shown.

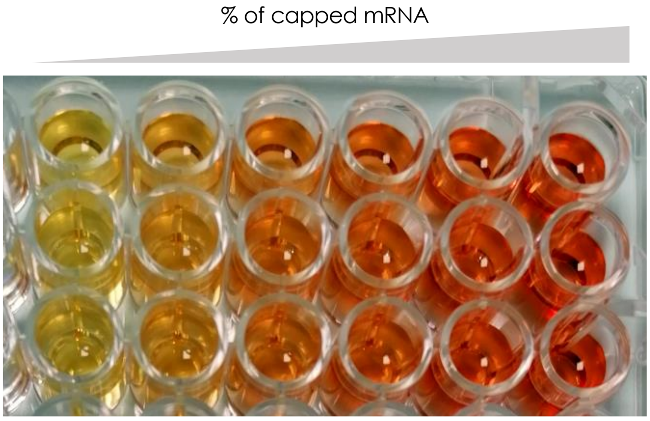

Figure S13. Visual inspection of the biosensor assay for $cRNA_1$ (Figure 3A) after 1.5 hours of reaction. Increasing percentage of capped mRNA from left to right. In each column three experimental replicates of the colorimetric assay are shown.

5. SUPPLEMENTARY REFERENCES

1. Niedzwiecka,A., Marcotrigiano,J., Stepinski,J., Jankowska-Anyszka,M., Wyslouch-Cieszynska,A., Dadlez,M., Gingras,A.C., Mak,P., Darzynkiewicz,E., Sonenberg,N., *et al.* (2002) Biophysical studies of eIF4E cap-binding protein: Recognition of mRNA 5′ cap structure and synthetic fragments of eIF4G and 4E-BP1 proteins. *J. Mol. Biol.*, **319**, 615–635.

2. Alcock,L.J., Perkins,M. V. and Chalker,J.M. (2018) Chemical methods for mapping cysteine oxidation. *Chem. Soc. Rev.*, **47**, 231–268.
